# Correlation Between Magnetic Resonance Imaging Biomarkers and Blood Biomarkers in Acute Ischemic Stroke

**DOI:** 10.3390/neurolint18100185

**Published:** 2026-09-27

**Authors:** Samirah A. Alameer, Reem T. Alturki, Alanood F. Alsaleh, Meaad M. Almusined

**Affiliations:** Department of Radiological Sciences, College of Applied Medical Sciences, King Saud University, Riyadh 11362, Saudi Arabiamalmusined@ksu.edu.sa (M.M.A.)

**Keywords:** magnetic resonance imaging biomarkers, blood biomarkers, acute ischemic stroke, AIS, biomarkers

## Abstract

**Background:** Acute ischemic stroke (AIS) is a major cause of mortality. Despite substantial advances in stroke diagnosis, investigation, and therapeutic management, the need for improved prognostic assessment remains an important clinical challenge. This study aimed to evaluate the role of combining magnetic resonance imaging (MRI)-derived and blood biomarkers in predicting functional outcomes in AIS at 3 months. **Methods:** A retrospective observational cohort study used data from Prince Mohd bin Naser Hospital in Saudi Arabia to integrate clinical variables and MRI-derived and blood biomarkers from adult patients with AIS. **Results:** The primary outcome, 3-month functional status, was assessed by the modified Rankin Scale (mRS) (good outcome: mRS < 3; poor outcome: mRS ≥ 3). The corrected analytical cohort included 100 patients. Among the 100 patients (median age: 61 years; male: 75.0%), 67 and 33 had good and poor 3-month outcomes, respectively. Compared with the good outcome group, the poor outcome group had significantly higher National Institutes of Health Stroke Scale (NIHSS) score at admission, which was the strongest discriminator between the two groups, and a trend toward a lower lesion’s mean apparent diffusion coefficient (ADC) that did not reach statistical significance. Multivariable analysis showed the best predictive performance (area under the curve (AUC): 0.934; cross-validated AUC: 0.906) with the base model consisting of admission NIHSS, lesion’s mean ADC, neutrophil percentage, and red cell distribution width; this cross-validated performance was not statistically distinguishable from a comparator model using NIHSS alone (CV-AUC: 0.898). Correlation analyses showed weak-to-moderate relationships between MRI-derived and blood biomarkers, with no correlations remaining significant after false discovery rate correction. **Conclusions:** Admission NIHSS score was the most powerful and consistent predictor of the 3-month outcome, while the incremental value of the multimarker model over NIHSS alone requires confirmation in larger, externally validated cohorts.

## 1. Introduction

Acute ischemic stroke (AIS) is one of the leading causes of mortality and long-term disability worldwide and remains a major public health challenge [1,2,3]. Despite substantial advances in stroke recognition, neuroimaging, thrombolytic therapy, mechanical thrombectomy, and rehabilitation, clinical outcomes continue to vary considerably among patients with apparently similar presentations [4,5,6]. Some individuals achieve a meaningful recovery, whereas others develop persistent neurological deficits despite timely diagnosis and treatment. This variability highlights the need for more accurate and clinically practical tools for early prognostic assessments [7,8].

Magnetic resonance imaging (MRI) plays a critical role in AIS evaluation as it provides detailed information on tissue injury, lesion burden, and spatial characteristics of ischemic damage [9]. Diffusion-weighted imaging (DWI) and apparent diffusion coefficient (ADC) values are particularly useful for characterizing the ischemic core and the severity of diffusion restriction [9,10]. Additional imaging parameters, including lesion area, intralesional ADC heterogeneity, and T2/FLAIR (fluid-attenuated inversion recovery) signal characteristics, may provide further insights into the infarct extent, tissue status, and lesion evolution [9,10]. However, MRI findings alone do not fully explain the wide variation in recovery across patients with AIS [10].

In parallel, blood-based biomarkers have gained increasing attention because they reflect the systemic biological responses to cerebral ischemia [7,8,11]. Functional outcomes after AIS are influenced by structural brain injury, inflammation, immune dysregulation, coagulation imbalance, hematological status, and metabolic stress [7,11,12]. Several of these processes can be assessed using routinely available laboratory parameters obtained during hospital admission. Biomarkers such as white blood cell count (WBC), neutrophils, lymphocytes, red cell distribution width (RDW), platelets, hemoglobin, glucose, international normalized ratio (INR), and activated partial thromboplastin time (APTT) are widely accessible and may provide important prognostic information [7,8,13].

Inflammatory activation is one of the earliest systemic responses to cerebral ischemia [12,14]. Elevated WBC and neutrophilia are associated with greater tissue injury, endothelial dysfunction, blood–brain barrier disruption, and poorer functional outcomes [14,15]. Lymphocyte alterations may reflect a post-stroke immune imbalance, and RDW has emerged as a low-cost marker associated with an unfavorable prognosis in several vascular conditions, including stroke [7,16]. Likewise, hemoglobin concentration may influence tissue oxygen delivery; admission hyperglycemia has been linked to more severe ischemic injury, and coagulation markers such as INR and APTT remain clinically relevant for interpreting stroke severity and risk profiles [8,11].

Current evidence suggests that no single biomarker can sufficiently explain AIS complexity [12,17]. A multidimensional approach that integrates imaging findings with blood biomarkers may provide a more comprehensive understanding of stroke pathophysiology and prognosis [10,12]. Accordingly, combining MRI-derived lesion characteristics with routine laboratory biomarkers may improve early risk stratification and enhance the prediction of functional outcomes after AIS [8,10,15]. However, research in this area needs more investigation.

This study aimed to evaluate the correlation between MRI biomarkers and routinely collected blood biomarkers in patients with AIS and their combined value in predicting 3-month functional outcomes. The findings of this study could help improve the prognosis and treatment of AIS.

## 2. Materials and Methods

### 2.1. Study Design and Setting

This retrospective observational cohort study used a structured dataset compiled from routinely collected clinical data, including MRI-derived biomarkers obtained from the hospital’s picture archiving and communication system (PACS) and blood biomarker measurements retrieved from electronic medical records for patients with AIS. All clinical data were collected from the Radiology Department at Prince Mohd bin Naser Hospital. The analyses were then performed using these previously collected patient data. Data extraction, cleaning, statistical analysis, and thesis reporting were conducted during the 2025/2026 academic year. Patient records were collected over the period from the beginning of 2023 to the end of 2024. Cases were not selected consecutively or randomly; rather, patients were selected based on the availability and completeness of the required data in the medical records, and only cases meeting the prespecified eligibility criteria with complete data were included.

### 2.2. Study Population

#### 2.2.1. Eligibility and Case Definition

The study population comprised adult patients with AIS who had available clinical assessments, MRI-derived biomarker measurements, and blood biomarker results recorded in the study dataset. Only records with sufficient data quality for the relevant analyses were retained.

- The inclusion criteria were adults aged 18 years or older with MRI-confirmed AIS within 24 h of symptom onset, with available clinical, MRI biomarker, and blood biomarker data, and follow-up outcome assessment at 3 months.
- The exclusion criteria were hemorrhagic stroke, preexisting inflammatory diseases, and/or preexisting neoplastic diseases. In addition, records with invalid outcome coding, duplicate entries, or non-interpretable key measurements were excluded from relevant analyses. Patients with more than one discrete ischemic lesion on ADC imaging were also excluded, to avoid inter-lesion overlap and to ensure standardized, single-lesion measurements; accordingly, only patients with a single ischemic lesion were included in the study.

#### 2.2.2. Final Analytical Sample

For outcome-based analyses, only patients with valid 3-month modified Rankin Scale (mRS) values were included. Records with missing or invalid mRS values (outside the accepted mRS range of 0–6) were excluded from the analyses involving functional outcome classification. The study population was restricted to patients who attended the 3-month follow-up visit and could therefore be assessed with the mRS at that time point; patients who died during the follow-up period were not included in the final analytical sample. Consequently, no patient in this cohort had a recorded mRS of 6 (death).

### 2.3. Data Sources and Study Variables

#### 2.3.1. Data Sources

Data was integrated into a unified analysis dataset from three structured Excel worksheets:

- Clinical data sheet: demographic and clinical severity variables, including age, sex, admission National Institutes of Health Stroke Scale (NIHSS) score, and 3-month mRS.
- Image biomarker sheet: MRI-derived lesion metrics and signal intensity measurements, including lesion area, lesion mean ADC, lesion ADC standard deviation (SD), lesion mean T2 signal intensity, and tissue-specific T2 signal intensity metrics (white matter and gray matter), when available.
- Blood biomarker sheet: Hematological and laboratory variables, including inflammatory markers (WBC, neutrophils, and lymphocytes), RDW, coagulation markers ( INR and APTT), glucose (mmol/L), platelets, and hemoglobin (g/dL), when available.

#### 2.3.2. Primary Outcome Variable

The primary outcome was the mRS-assessed functional outcome at 3 months. For binary outcome modeling, the mRS was dichotomized as follows:

- Good outcome: mRS < 3.
- Poor outcome: mRS ≥ 3.

This dichotomization has been consistently used in descriptive comparisons, univariate analyses, and multivariable predictive modeling [18,19].

#### 2.3.3. Imaging Biomarkers and MRI Quantification

The MRI biomarker variables were derived from the image biomarker worksheet and included lesion-level measurements and tissue signal intensity parameters. Particular focus was placed on mean ADC, T2 signal intensity, and lesion area because of their biological relevance in acute ischemic injury.

The ADC values were interpreted using the unit ×10^−6^ mm^2^/s, and this unit was retained in the tables, figures, and statistical reporting throughout the study to ensure consistency with the measurement source. Where lesion location information (white matter vs. gray matter) was available, it was harmonized to support the subgroup descriptions and location-based comparisons.

#### 2.3.4. MRI Biomarker Extraction and Quantitative Variable Handling

MRI examinations were performed as part of the routine acute stroke evaluation in the radiology department of Prince Mohd bin Naser Hospital. Quantitative MRI-derived measurements were obtained by placing regions of interest (ROIs) on the existing clinical MRI images retrieved from the patients’ imaging records as part of the present study. The image biomarkers analyzed in this study included the lesion mean ADC, lesion ADC standard deviation, lesion mean T2 signal intensity, white matter mean T2 signal intensity, gray matter mean T2 signal intensity, and lesion area. These variables were treated as structured quantitative measurements imported from the image biomarker worksheet (Figure 1 and Figure 2).

MRI examinations were performed within 24 h of symptom onset using a 1.5 Tesla scanner (Siemens Healthineers, Erlangen, Germany). The imaging protocol included T1-weighted, T2-weighted, fluid-attenuated inversion recovery (FLAIR), and diffusion-weighted imaging (DWI) sequences. DWI was acquired using b-values of 0 and 1000 s/mm^2^, from which apparent diffusion coefficient (ADC) maps were generated. Regions of interest (ROIs) were placed manually on the ADC maps within the most clearly demarcated portion of the ischemic lesion; “lesion area” therefore refers to the area of the manually placed ROI rather than the total infarct volume. Image review and quantitative measurements were performed using a dedicated clinical viewing and measurement workstation; ADC and T2 signal intensity values were recorded directly from the ROI without age- or tissue-based normalization, and were interpreted with reference to previously published normal ADC ranges (Table 1) [20].

Region-of-interest (ROI) placement for quantitative ADC and T2 signal intensity measurement (Figure 1 and Figure 2) was performed by two of the authors, S.A. Alameer, MSc (10 years of experience), and M.A. Almusined, PhD (14 years of experience), on the existing clinical MRI images retrieved from the patients’ imaging records, using a consensus reading approach. Readers were not formally blinded to the patients’ 3-month functional outcome at the time of ROI placement, and inter-reader agreement was not formally assessed.

#### 2.3.5. Blood Biomarkers

Blood biomarker variables were selected based on their availability in the dataset and clinical plausibility of stroke pathophysiology. These included inflammatory markers (e.g., WBC, neutrophils, and lymphocytes), hematological descriptors (e.g., RDW, platelet count, and hemoglobin), coagulation markers (e.g., INR and APTT), and metabolic biomarkers, including glucose (mmol/L).

Hemoglobin (g/dL) was explicitly included in the final analysis workflow and assessed by group comparisons, correlation analyses, and model expansion analyses to evaluate whether it added prognostic information beyond the base model. Advanced blood biomarkers were not included in this dataset.

Several advanced blood biomarkers were unavailable in this retrospective dataset and were excluded from the final analysis. These include glial fibrillary acidic protein (GFAP), Neurofilament light chain (NfL), neuron-specific enolase (NSE), Ubiquitin Carboxy-terminal Hydrolase L1 (UCH-L1), and Matrix Metalloproteinase-9 (MMP-9), which have been investigated as markers of astroglial injury, axonal damage, neuronal injury, and blood–brain barrier disruption in stroke research [12,21]. Although such biomarkers are of growing interest in Saudi Arabia, they are still considered investigational rather than routine components of the current hospital datasets.

#### 2.3.6. Blood Sampling, Laboratory Processing, and Biomarker Extraction

Blood biomarker values were obtained from routine laboratory investigations requested during acute stroke care at the Prince Mohd bin Naser Hospital. Laboratory variables were analyzed as recorded in the study dataset, and measurement units were retained, as reported by the hospital laboratory, to preserve clinical interpretability and consistency with source records. Because biomarker levels may vary over time in AIS, the timing relative to symptom onset, hospital admission, and MRI acquisition was considered during interpretation whenever timing information was available in the records.

### 2.4. Data Management and Preprocessing

#### Data Integration and Row Matching

Three worksheets (clinical, imaging, and blood biomarkers) were merged row-wise after patient-level row matching. Because row-order mismatches can introduce significant analytical errors, a dedicated data quality review was performed prior to the final analysis. The corrected version of the workbook was used for all final tables and figures.

Post-correction verification included consistency checks across worksheets using duplicated fields (e.g., age and sex) to confirm successful row matching.

### 2.5. Data Cleaning and Variable Harmonization

Data cleaning was performed before analysis to ensure internal consistency and valid statistical computations. The following preprocessing rules were applied:

- Non-numeric entries in numeric variables were coerced by missing values during data import for statistical analysis.
- The mRS values were validated, and values outside the accepted range (0–6) were treated as invalid and excluded from the outcome analyses.
- Categorical text fields were standardized (e.g., sex coding and lesion location labels) to remove spacing and case inconsistencies.
- Autogenerated empty columns (e.g., unnamed spreadsheet columns) were excluded from image biomarker analysis.
- A derived binary outcome variable (poor_mrs) was created from the 3-month mRS.
- A derived lesion-location binary variable (white matter = 1, gray matter = 0) was created when location information was available.

### 2.6. Missing Data Handling

Missing data were handled according to the type of data analyzed. For descriptive and correlation analyses, pairwise available case analysis was used (i.e., each calculation used all records with non-missing values for the variables under comparison). For multivariable predictive modeling, missing predictor values were imputed using median imputation within a machine learning pipeline to avoid unnecessary case deletions and preserve the reproducibility of model fitting.

### 2.7. Statistical Analysis

Statistical analysis was conducted to describe the cohort, compare biomarker distributions across outcome groups, examine relationships between MRI and blood biomarkers, and evaluate multivariable predictions of poor functional outcomes. The analytical workflow was implemented in Python using a reproducible script-based pipeline.

### 2.8. Statistical Rationale for Test Selection

A statistical approach was selected to match the structures of available multimodal datasets. Acute stroke biomarker data often show non-normal distributions, outliers, and unequal variance across outcome groups; therefore, non-parametric summaries (median and interquartile range) and the Mann–Whitney–U test were used for primary continuous-variable comparisons.

Spearman’s rank correlation was used for MRI–blood biomarker relationship analysis because it assesses monotonic associations and is less sensitive to non-normal and influential observations than Pearson’s correlation. Logistic regression was selected for prognostic modeling because the primary endpoint was binary (poor versus good 3-month functional outcomes).

### 2.9. Descriptive Statistics

Continuous variables were summarized using median and interquartile range (IQR; Q1–Q3) because several biomarkers were not assumed to be normally distributed. Categorical variables were summarized as frequencies and percentages.

### 2.10. Group Comparisons by Functional Outcome

Patients were grouped into good (mRS < 3) and poor (mRS ≥ 3) outcomes. Group comparisons were performed as follows.

- Mann–Whitney–U test was used for continuous variables (non-parametric comparison between two independent groups).
- The chi-square test (or Fisher’s exact test when the expected cell counts were small) was used for categorical variables, where applicable.

Two-sided *p*-values were reported. Univariate ranking tables were generated to identify variables that showed the strongest association signals based on *p*-value ordering.

### 2.11. Correlation Analysis Between MRI and Blood Biomarkers

To investigate the relationships between MRI-derived variables and blood biomarkers, Spearman’s rank correlation coefficients (rho) were computed for a priori-selected pairs of variables based on biological plausibility, availability in the corrected dataset, and relevance to the study objectives.

For each pairwise comparison, the following were reported when the data were sufficient: sample size (N), Spearman’s rho, and *p*-value. Correlation findings were presented using ranked tables and a lower-triangle heatmap to reduce mirrored redundancy in the correlation matrix.

To improve the interpretability of the thesis tables, a strength-category notation was added based on the absolute value of Spearman rho (|rho|). The categories are defined as follows:

- Very strong: |rho| ≥ 0.80.
- Strong: 0.60 ≤ |rho| < 0.80.
- Moderate: 0.40 ≤ |rho| < 0.60.
- Weak: 0.20 ≤ |rho| < 0.40.
- Very weak/negligible: |rho| < 0.20.

These strength categories only describe the magnitude of the effect and do not replace formal statistical significance testing.

### 2.12. Multivariable Predictive Modeling

The primary predictive endpoint for multivariable modeling was a poor 3-month functional outcome (mRS ≥ 3). Logistic regression models were used to estimate the probability of poor outcomes using a combination of clinical, MRI, and blood biomarker predictors.

The four base-model predictors (admission NIHSS score, lesion mean ADC, neutrophil percentage, and RDW) were specified a priori, before univariate analysis, based on their established biological plausibility and prior literature evidence as candidate prognostic markers in acute ischemic stroke; they were not selected post hoc on the basis of univariate ranking. A base model was therefore defined using these prespecified clinically and analytically relevant predictors available in the corrected dataset. Model expansion analyses were then performed to evaluate whether adding hemoglobin and/or age improved the predictive performance.

Predictive modeling was implemented using a standardized pipeline comprising:

- Median imputation for missing predictor values.
- Feature scaling (standardization).
- Logistic regression classifier.

The model performance was assessed using both the apparent areas under the receiver operating characteristic curve (AUC) and 5-fold stratified CV-AUC. Cross-validated predicted probabilities were used to generate receiver operating characteristic (ROC) curves for model comparison.

### 2.13. Cutoff Optimization and Diagnostic Performance Indices

For the best-performing model (based on the cross-validated AUC), an optimal probability cutoff was selected using the Youden index applied to the pooled out-of-fold predicted probabilities from the 5-fold stratified cross-validation (i.e., each patient’s prediction from the fold in which they were held out, combined across all folds before cutoff selection). Diagnostic performance indices, including sensitivity, specificity, positive predictive value (PPV), negative predictive value (NPV), and overall accuracy, were then calculated at the selected cutoff. Model calibration was additionally assessed using the cross-validated predicted probabilities, by estimating the calibration slope and intercept from a logistic regression of the observed outcome on the logit of the predicted probability, by calculating the Brier score, and by the Hosmer–Lemeshow goodness-of-fit test.

### 2.14. Multivariable Effect Estimates

To improve the clinical interpretability of the multivariable models, logistic regression effect estimates were reported for selected model specifications as odds ratios (ORs) with 95% confidence intervals (CIs), and corresponding predictor-specific *p*-values were estimable. These estimates were used to complement the model discrimination metrics (AUC/CV-AUC) and describe the direction and magnitude of the association between the candidate predictors and poor 3-month functional outcomes.

### 2.15. Significance Threshold and Statistical Software

All statistical tests were two-sided, and statistical significance was set at *p* < 0.05, unless otherwise specified. The analyses were performed using Python 3.14. using standard scientific computing and statistical libraries, including pandas, NumPy, SciPy, scikit-learn, statsmodels, matplotlib, and Python-docx for reproducible reporting outputs.

All methodological details relevant to this study are provided in the main manuscripts.

### 2.16. Ethical Considerations

This retrospective observational cohort study used patient-derived clinical, imaging, and laboratory data from Prince Mohd bin Naser Hospital, part of the Jazan Health Cluster, Saudi Arabia. Ethical approval was obtained from the Jazan Health Cluster Ethics Committee, Saudi Arabia (National Registration Number: NCBE-KACST, KSA: H-10-Z-141) under Approval No. 25175 (expedited review) on 21 December 2025.

The ethics approval documentation stated that the study information would be obtained from patient files without access to direct personal identifiers. Accordingly, patient confidentiality was maintained through data de-identification, restricted access to study files, and the use of analytical datasets without direct identifiers. Administrative approval from the study site was also obtained as stipulated by the committee.

## 3. Results

This section presents the results of a comprehensive integrated analysis of MRI-derived and circulating blood biomarkers in AIS. The findings were structured according to the study objectives, encompassing cohort characterization, comparative analyses of 3-month functional outcomes, correlation analyses between MRI parameters and blood biomarkers, and evaluation of prognostic performance using multivariable models. Taken together, these analyses provide a robust quantitative framework for investigating the potential incremental clinical utility of combining imaging and blood-based biomarkers to enhance outcome prediction after AIS.

### 3.1. ADC Unit Documentation and Reference Interpretation Framework

ADC measurements were interpreted and reported in units of ×10^−6^ mm^2^/s, consistent with the reference image and the measurement convention adopted in this study (Table 1). This unit was consistently used throughout the Results section to present lesion ADC values, between-group comparisons, and association analyses, ensuring methodological consistency and accurate clinical-radiological interpretation.

### 3.2. Data Quality

The corrected workbook included 100 records. During final data verification for this revision, one record with an implausible 3-month mRS value (entered as 23, outside the valid 0–6 range) was cross-checked against source documentation and corrected to the true value of 3; all 100 records therefore had valid 3-month mRS values and were retained for outcome-based analyses (Table 2). Patient selection is summarized in Figure 3.

### 3.3. Demographic Characteristics

As shown in Table 3, the analyzed cohort comprised 100 patients with valid 3-month mRS data. The median age of the cohort was 61.0 years (IQR: 50.8–74.0), and the study population was predominantly male, comprising 75 males (75.0%) and 25 females (25.0%). When stratified by the 3-month functional outcomes, patients with poor outcomes tended to be older than those with good outcomes (median: 65.0 years vs. 59.0 years), although this difference did not reach statistical significance (*p* = 0.165). Sex distribution was similar between the two groups, with males representing 76.1% of the good outcome group and 72.7% of the poor outcome group (*p* = 0.807). Overall, these findings indicated that the cohort was predominantly male, and no statistically significant demographic differences were observed between the outcome groups.

### 3.4. Group Comparisons by 3-Month Functional Outcome

Continuous variables were compared between the good- and poor-outcome groups using the Mann–Whitney–U test. Table 4 and Table 5 summarize the principal differences in baseline characteristics and biomarker measurements between the two groups and identify the variables potentially associated with the 3-month functional outcome.

Table 4 shows that patients with poor 3-month functional outcome (mRS ≥ 3) had a significantly higher admission NIHSS score than those with good outcome (median 9.0 vs. 4.0, *p* < 0.001). The lesion’s mean ADC showed a trend toward lower values in the poor-outcome group (median: 455.0 vs. 516.0 × 10^−6^ mm^2^/s), without reaching statistical significance (*p* = 0.072). No statistically significant between-group differences were observed for age, lesion area, lesion ADC SD, lesion mean T2 signal intensity, neutrophil percentage, lymphocyte percentage, RDW, hemoglobin, glucose, INR, or APTT (all *p* > 0.05), although several variables showed borderline trends, including lesion location (*p =* 0.060), lesion mean ADC (*p* = 0.072), neutrophils (*p* = 0.074), lesion area (*p* = 0.078), and RDW (*p* = 0.081).

### 3.5. Ranked Univariate Comparison

Table 5 shows the ranking of the variables by univariate *p*-values. The NIHSS score at admission was the strongest discriminator between the outcome groups, with significantly higher values in the poor-outcome group than in the good-outcome group (median: 9.0 vs. 4.0; *p <* 0.001). The lesion’s mean ADC showed a borderline trend toward lower values in the poor-outcome group (median: 455.0 vs. 516.0 × 10^−6^ mm^2^/s, *p* = 0.072), consistent with greater diffusion restriction, although this did not reach conventional statistical significance. Several additional variables demonstrated borderline associations with the 3-month outcomes, including lesion location (*p* = 0.060), neutrophil percentage (*p* = 0.074), lesion area (*p* = 0.078), and RDW (*p* = 0.081). In contrast, age, T2 signal intensity metrics, glucose level, lymphocyte percentage, red blood cell (RBC) count, and hemoglobin level were not significantly different between the two groups in the ranked univariate analysis (all *p* > 0.05).

### 3.6. Descriptive and Comparative Analysis

This section presents the descriptive and comparative analyses of the study cohort and provides an initial statistical overview of the clinical, MRI-derived, and blood biomarker variables included in the integrated dataset. Descriptive statistics summarize the distributions of key variables, while outcome-based comparisons examine differences between patients with good and poor 3-month functional outcomes. These analyses provided a statistical context for subsequent correlation analyses and multivariable prognostic modeling.

The histogram in Figure 4 shows the distribution of functional outcomes and confirms sufficient variability for binary outcome modeling. The dichotomization threshold used in this section was mRS < 3 (good outcome) versus mRS ≥ 3 (poor outcome). The distribution of 3-month mRS scores showed a clear concentration in the lower disability categories, with the highest frequency observed at mRS 1, followed by mRS 2 and mRS 0. This indicates that most patients achieved favorable functional outcomes at 3 months. The frequency declined progressively across higher mRS categories, with fewer patients demonstrating moderate-to-severe disability (mRS 3–5), whereas those with mRS 6 were absent or extremely rare. Overall, the distribution suggests that the study cohort was predominantly characterized by good functional recovery, although a small proportion of patients had clinically relevant disabilities at follow-up.

Figure 5 shows an upward shift in admission NIHSS scores in the poor-outcome group, supporting the established role of baseline stroke severity as a major prognostic determinant.

Figure 6 shows lower mean ADC values in lesions from the poor-outcome group, consistent with stronger diffusion restriction and more severe acute ischemic injury. The ADC values were interpreted with reference to the tissue ranges listed in Table 1.

Figure 7 compares neutrophil distributions between good- and poor-outcome groups and visually supports the borderline-to-significant inflammatory signal observed in the univariate and multivariable analyses (Table 3, Table 4, Table 6 and Table 7).

Figure 8 shows a partial overlap in RDW distributions between the good- and poor-outcome groups, consistent with the borderline univariate difference observed in Table 3 (*p* = 0.081) and the lack of independent significance after multivariable adjustment (Table 6 and Table 7).

Figure 9 shows a substantial overlap in hemoglobin values between the good- and poor-outcome groups, consistent with the non-significant univariate comparison and the absence of an independent effect in the multivariable models.

Multivariable predictive models, logistic regression estimates, and ROC-derived cutoff performance.

Multivariable logistic regression models were developed to evaluate the combined prognostic value of selected clinical, MRI, and blood biomarker variables for predicting the 3-month functional outcomes. Model performance was assessed using the predicted probabilities obtained from 5-fold stratified cross-validation, and discrimination was quantified using ROC analysis and AUC. The corresponding cross-validated ROC curves are shown in Figure 10.

As shown in Table 6, the base model (admission NIHSS score, lesion mean ADC, neutrophils, and RDW) demonstrated excellent discriminatory performance with an apparent AUC of 0.934 and the highest cross-validated performance (CV-AUC: 0.906) among the evaluated models. Adding hemoglobin to the base model produced a slight reduction in performance (apparent AUC: 0.932, CV-AUC: 0.896). The extended model, which also included age, achieved a comparable apparent AUC (0.937) but a lower cross-validated AUC (0.892), suggesting no improvement in the generalizable predictive performance compared with the base model. To evaluate the incremental value of the multimarker approach, a comparator model using admission NIHSS score alone was also evaluated (apparent AUC: 0.906; CV-AUC: 0.898). The CV-AUC of the base model was only marginally higher than that of the NIHSS-alone model, and a bootstrap-based comparison indicated that this difference was not statistically significant (mean difference: 0.008; 95% CI: −0.036 to 0.051), suggesing that any incremental discriminative value of the multimarker model over NIHSS alone should be interpreted cautiously given the sample size.

Multivariable logistic regression estimates for the base model are presented in Table 7. In this model, admission NIHSS score was a strong independent predictor of poor outcome (OR: 2.57, 95% CI: 1.70–3.87, *p* < 0.001), and neutrophil percentage was also significantly associated with poor outcome (OR: 1.07, 95% CI: 1.02–1.12, *p* = 0.010). Lesion’s mean ADC showed a borderline inverse association with poor outcome (OR: 0.99, 95% CI: 0.99–1.00, *p* = 0.069), while RDW was not statistically significant (OR: 1.12, 95% CI: 0.91–1.38, *p* = 0.278). Variance inflation factors for all four predictors were close to 1.0 (range: 1.02–1.04), indicating no evidence of multicollinearity among the base model predictors. The magnitude of the NIHSS odds ratio warrants explanation: an OR of 2.57 reflects the estimated multiplicative change in the odds of poor outcome per single 1-point increase in admission NIHSS score, holding the other three predictors constant. Because the admission NIHSS scores of the good- and poor-outcome groups were largely non-overlapping in this cohort (interquartile range 2.0–5.0 vs. 6.0–12.0; standard deviation 3.6 points across the full sample), the fitted logistic slope on this variable is steep, and per-point extrapolation over several points (e.g., a hypothetical 5-point increase corresponding to 2.57^5^ ≈ 112-fold odds) should not be interpreted literally as an isolated, generalizable per-point risk; rather, it summarizes a strong but sample-specific discriminative slope that requires external validation before clinical extrapolation.

After adding hemoglobin, the regression estimates shown in Table 8 remained broadly consistent with the base model. Admission NIHSS score remained strongly associated with poor outcome (OR: 2.55, 95% CI: 1.69–3.85, *p* < 0.001), and neutrophil percentage retained statistical significance (OR: 1.06, 95% CI: 1.01–1.12, *p* = 0.022). The lesion’s mean ADC remained borderline (OR: 0.99, 95% CI: 0.99–1.00, *p* = 0.066), whereas RDW and hemoglobin were not significant predictors in the adjusted model (RDW: *p* = 0.382; hemoglobin: *p* = 0.719).

As the base model showed the best cross-validated discrimination, it was selected as the best-performing model for threshold optimization. As presented in Table 9, the optimal probability cutoff determined by the Youden index was 0.382, yielding a sensitivity of 81.8%, specificity of 89.6%, PPV of 79.4%, NPV of 90.9%, and overall accuracy of 87.0%. The model demonstrated good discriminative ability in this cohort; however, given the limited sample size and the lack of a statistically significant advantage over the NIHSS-alone comparator, this level of performance should be considered hypothesis-generating and requires external validation before its clinical application can be considered. Calibration of the cross-validated predicted probabilities was satisfactory: the calibration slope was 1.12 (1.0 indicates perfect calibration), the calibration intercept was 0.07 (0 indicates no systematic over- or under-prediction), the Brier score was 0.099, and the Hosmer–Lemeshow goodness-of-fit test showed no evidence of miscalibration (χ^2^ = 10.53, df = 8, *p* = 0.230).

Overall, the base model showed the best cross-validated discrimination and was therefore selected for cutoff optimization and ROC curve visualization (Table 6, Table 7, Table 8 and Table 9) (Figure 10).

As illustrated in Figure 10 and summarized in Table 6, the base model demonstrated the best cross-validated discriminatory performance, supporting its selection for cutoff optimization (Table 9). ROC analysis of the 5-fold cross-validated predicted probabilities demonstrated good discriminative performance for all evaluated models. The base model, including the NIHSS score, ADC, neutrophil percentage, and RDW, achieved the highest CV-AUC (0.906), followed closely by a NIHSS-alone comparator model (CV-AUC: 0.898). Adding hemoglobin resulted in a marginally lower CV-AUC (0.896), whereas the extended model, including age, base variables, and hemoglobin, showed a slightly lower CV-AUC (0.892). All the models performed substantially better than chance, as reflected by ROC curves that lie well above the reference diagonal. Overall, the findings suggested that the base model provided the most parsimonious prediction model; however, its cross-validated performance was not statistically distinguishable from that of NIHSS alone in this sample, and the incremental value of the multimarker approach should be confirmed in larger, externally validated cohorts.

### 3.7. Spearman’s Rank Correlation Analysis Between MRI and Blood Biomarkers

Spearman’s rank correlation was computed between the selected MRI/clinical variables and blood biomarkers. A strength category star notation was added to facilitate the interpretation of the effect magnitude based on the absolute value of Spearman’s rho. Because a large number of pairwise correlations were tested, the Benjamini–Hochberg–procedure was applied to control the false discovery rate (FDR) across the full family of 63 MRI/clinical–blood–biomarker comparisons actually performed (the 56 general comparisons summarized in Table 10 plus the 7 hemoglobin-specific comparisons summarized in Table 11), and the resulting FDR-adjusted q-values are reported alongside the nominal *p*-values in both tables.

As shown in Figure 11, most Spearman’s rank correlations among the selected clinical, MRI, and blood biomarker variables were weak to moderate in magnitude, indicating limited multicollinearity across the integrated dataset. The strongest inverse correlation in the displayed matrix was between hemoglobin and RDW (rho = −0.40), consistent with the expected hematologic relationship. A weak inverse correlation was also noted between hemoglobin and neutrophils (rho = −0.26), while weak positive correlations were observed between glucose and neutrophils (rho = 0.25) and between lesion area and admission NIHSS score (rho = 0.25). MRI-related variables showed weak correlations with the blood biomarkers in this matrix. For example, the lesion’s mean ADC demonstrated weak inverse correlations with the admission NIHSS score (rho = −0.18) and lesion area (rho = −0.23), whereas the lesion mean T2 signal intensity showed minimal pairwise correlations with most of the included variables (rho values near zero). Overall, Figure 11 supports the use of multivariable modeling, indicating that the candidate predictors were not strongly correlated with one another.

Figure 12 shows the distribution of lesion’s mean ADC values by lesion location category (gray matter vs. white matter), providing an anatomical context for lesion-level ADC variability prior to multivariable modeling.

### 3.8. Multi-Panel Relationship

Figure 13 presents scatterplots with fitted linear trend lines and in-panel statistics (R^2^, slope *p*-value, Spearman rho, and Spearman *p*-value). Overall, most associations appeared weak and dispersed, consistent with the low-to-moderate correlation magnitudes reported in Table 10.

Figure 14 shows the relationships between lesion metrics (ADC, T2 signal intensity, and lesion area) and inflammatory markers (WBC, neutrophils, and RDW). The panels visually support the generally weak MRI–inflammatory–biomarker associations summarized in Table 10 while helping to identify directionality and potential outlier influences across pairings.

Figure 15 shows weak and inconsistent associations between hemoglobin and MRI lesion metrics/admission NIHSS, consistent with the non-significant correlations and lack of an independent predictive contribution in the multivariable models, as detailed in Table 11.

### 3.9. Integrated Interpretation

The NIHSS score at admission remained the strongest predictor of the 3-month functional outcome. The lesion’s mean ADC retained biologically meaningful interpretive value when reported in ×10^−6^ mm^2^/s and interpreted against reference tissue ranges. Among the blood biomarkers, inflammatory markers, particularly neutrophils (and to a lesser extent, RDW), were more informative than hemoglobin in this dataset. Hemoglobin was included in the group comparisons, correlation analyses, and multivariable model testing; however, it did not show a clear independent contribution to outcome discrimination.

## 4. Discussion

This study assessed the integrated prognostic value of clinical severity, MRI-derived lesion metrics, and routine blood biomarkers in predicting 3-month functional outcomes after AIS. The admission NIHSS score was the strongest and most stable predictor of poor functional outcome, while the lesion mean ADC provided biologically meaningful imaging information with borderline independent significance after multivariable adjustment. Blood biomarkers, such as neutrophil percentage, emerged as the most informative and independently significant factors, whereas RDW showed only a borderline sign, and hemoglobin did not add meaningful prognostic information as a separate factor. Furthermore, the base multivariable model incorporating NIHSS, lesion mean ADC, neutrophils, and RDW achieved the best internal discriminative performance, supporting the value of the parsimonious multimarker method over reliance on a single predictor.

The finding that the admission NIHSS score was the strongest predictor of the 3-month outcome is consistent with previous stroke study. In our study, the NIHSS score was higher in the poor outcome group and remained strongly correlated with poor outcomes after multivariable adjustment. This result is clinically plausible because the NIHSS captures the immediate neurological burden of stroke across multiple domains, including motor-, language-, sensory-, and consciousness-related deficits. Previous studies have consistently shown that baseline NIHSS is among the most powerful early predictors of 90-day functional status, and this prediction improves when early neurological changes are incorporated into prognostic models [18].

Regarding imaging biomarkers, the mean ADC of lesions was very low in patients with poor outcomes in the univariate comparison and remained borderline significant after multivariable adjustment. Lower ADC values in acute ischemic lesions generally reflect more severe diffusion restriction and more pronounced cytotoxic edema, which, in turn, suggest greater tissue injury and lower tissue viability. MRI-based studies support the prognostic relevance of ADC-derived characteristics in AIS and indicate that diffusion-based lesion characterization can improve the prediction of functional outcomes by aggregating clinical variables [19]. Beyond the lesion mean ADC, this study also captured intralesional ADC heterogeneity through the lesion ADC standard deviation (Lesion ADC SD), which was included as a separate variable in the univariate comparisons (Table 4 and Table 5); this metric showed a borderline association with outcome (*p* = 0.112) and is conceptually related to, though simpler than, formal texture-analysis approaches. Mean ADC and its within-lesion SD were therefore not the sole imaging descriptors examined in this study, and future work could extend this approach using full radiomic texture analysis.

Nevertheless, the borderline-adjusted significance of the lesion’s mean ADC should be interpreted carefully rather than being overstated. This pattern does not imply that ADC lacks value; rather, it likely reflects a partial overlap between imaging and clinical severity, as patients with more severe lesions often also have higher NIHSS scores. This may also reflect the limited statistical power and use of a single summary metric that cannot fully capture lesion heterogeneity. Therefore, the ADC appears to be best interpreted as a complementary imaging marker rather than a replacement for baseline clinical assessment. This interpretation aligns with contemporary stroke prognostic reviews in which imaging features are most useful when integrated with clinical and laboratory variables [19].

One of the most important findings of this study was the independent contribution of neutrophil percentage. Although the univariate association was only borderline, neutrophils remained significant in both multivariable models, suggesting that the inflammatory signals captured by neutrophils contributed to prognostic information beyond baseline stroke severity and lesion ADC. This result is biologically credible. Neutrophils are among the immune cells that are activated early after ischemic stroke and are involved in blood–brain–barrier disruption, edema formation, thrombo-inflammatory amplification, and secondary tissue injury [22]. Similarly, clinical studies have shown that neutrophil-related inflammatory indices are associated with poor 3-month outcomes and higher post-stroke risk, supporting the interpretation that early inflammation is an important aspect of recovery.

Additional useful findings showed that the neutrophil percentage, even without a composite inflammatory ratio, could provide clinically relevant prognostic information. This is important because the neutrophil percentage is inexpensive, routinely present, and easy to explain in daily practice. At the same time, neutrophils are not disease-specific markers, and their levels may also be influenced by infection, physiological stress, or comorbid inflammatory conditions. Nevertheless, their persistence after adjustment in this study suggests that they capture a meaningful pathophysiological border of AIS rather than a nonspecific basal variation.

RDW was slightly higher in the poor-outcome group than in the good-outcome group and showed a borderline univariate association; however, it did not remain independently significant after multivariable adjustment. This pattern is inconsistent with that reported in the current literature. Recent evidence suggests that an elevated RDW is associated with poor functional outcomes and mortality after AIS; however, the magnitude and consistency of this association vary across studies and populations [23]. The RDW is biologically attractive because it may reflect anisocytosis, chronic inflammation, oxidative stress, impaired erythropoiesis, or underlying systemic vulnerability. However, the same influences also reduce the specificity. Accordingly, the present results support a restrained interpretation: RDW may serve as an adjunctive marker; however, in this cohort, it did not demonstrate a stable independent prognostic value.

In contrast, hemoglobin showed no meaningful difference between the good-and poor-outcome groups, no significant correlation with MRI lesion metrics or baseline NIHSS scores, and no independent contribution after model adjustment. Furthermore, adding hemoglobin to the base model did not improve cross-validated performance. This result is valuable because it prevents overinterpretation of a routinely measured but clinically inconsistent marker. The literature on hemoglobin and anemia in patients with AIS remains unclear. Some reviews suggest that anemia may be associated with worse outcomes, especially in selected groups, such as patients with large-vessel occlusion, where oxygen delivery constraints may be of greater biological importance [24]. However, other studies have shown that hemoglobin concentration alone does not independently predict the 3-month functional outcome after accounting for neurological severity and comorbidities [25]. The current results align closely with the latter.

Another important observation in the present study was that pairwise correlations among the candidate predictors were generally weak to moderate, with no evidence of strong multicollinearity. This supports the rationale for building integrated multivariable models because the included predictors did not simply repeat the same biological information. Instead, they present different dimensions of the disease process, including baseline neurological severity, structural lesion injury, and systemic inflammatory responses. Conceptually, this is one of the key strengths of the present study because stroke outcomes are multifactorial and therefore unlikely to be adequately explained by any single domain.

The model performance analysis also deserves attention. The base model showed the best cross-validated discrimination, whereas adding hemoglobin resulted in a slight reduction in performance. This is methodologically important because it indicates that expanding a model with additional variables does not necessarily improve the prediction, even when these variables are biologically plausible. Practically, the base model appeared to offer a balance between interpretability and performance for the present dataset. This finding is consistent with the general prediction model protocol, which emphasizes parsimony and the need to assess performance beyond the derivation sample, rather than maximizing the apparent performance within the original dataset alone [26]. It should also be acknowledged that established stroke prognostic scores, such as the PLAN score, the iScore, and the ASTRAL score, [27,28,29] have reported comparable or in some validation cohorts higher discrimination (AUC values ranging from approximately 0.82 to 0.92 for PLAN, 0.72 to 0.88 for iScore, and 0.83 to 0.87 for ASTRAL across different populations), and the present base model’s cross-validated AUC (0.906) sits within this same range rather than clearly exceeding it. Similarly, the earlier Ischemic Stroke Survival Score of Williams and Jiang [30] reported a c-statistic of 0.86 for 1-year survival prediction, and a recent study by Kulikova et al. [31] identified a broadly overlapping set of predictors (including admission neurological severity and hematologic parameters) in a larger AIS cohort (n = 254) from a different population. Consistent with this, the present model’s performance was not statistically distinguishable from a simple NIHSS-alone comparator in this cohort. Accordingly, the principal contribution of this study should be understood as an internal validation and confirmation of previously described prognostic associations—admission stroke severity, diffusion-restriction imaging findings, and neutrophil-driven inflammation—within a Saudi Arabian AIS cohort in which these markers had not previously been evaluated together, rather than as the introduction of a novel prognostic model intended to outperform existing validated scores.

The present study provides the clinically relevant conclusion that early prognosis after AIS is better approached through an integrated assessment rather than single-marker reasoning. The baseline NIHSS score was the dominant anchor for prognosis, but the lesion’s mean ADC and neutrophils provided complementary information on tissue injury and inflammatory biology. RDW may be incorporated in a weak pattern in some settings, whereas hemoglobin appears to be less informative in this collective work. This integrated interpretation is more consistent with contemporary stroke medicine than the reductionist one-biomarker method.

This study has several strengths. First, it did not rely on a single predictor but instead assessed a combined framework integrating clinical severity, MRI-derived lesion characteristics, and routine blood biomarkers. This design is more biologically realistic and clinically relevant than a single-domain analysis. Second, it used cross-validated model evaluation rather than relying solely on apparent in-sample performance. By comparing candidate models using a cross-validated AUC, this study adopted a more rigorous approach to model selection and reduced the risk of overvaluing improvements in discrimination. Third, the inclusion of routinely available markers, such as neutrophils, RDW, and hemoglobin, enhanced the potential translational value of the findings. These attributes are inexpensive and can be implemented within established hospital practices, thereby facilitating future clinical applications.

This study also has several limitations. Although the study was sufficient to detect strong signals, the sample size may still have been moderate for the stability estimation of weaker effects in multivariable models. With 33 patients experiencing the poor-outcome event and 4 predictors in the base model, the events-per-variable (EPV) ratio was approximately 8.3, which is close to but below the commonly recommended minimum of 10; the EPV for the hemoglobin-augmented model (5 predictors, EPV ≈ 6.6) and the age-extended model (6 predictors, EPV ≈ 5.5) fell further below this threshold, indicating an increasing risk of overfitting for the expanded models. This may partly explain why the lesion’s mean ADC and RDW remained borderline rather than exactly significant. Second, the evaluated biomarkers were assessed as baseline or single-time-point variables. Inflammatory and hematologic responses after stroke are dynamic, and serial measurements may more accurately capture the evolving pathophysiology and improve prognostic discrimination. Third, ROI-based ADC and T2 signal intensity measurements were obtained by consensus reading between two authors without formal blinding to the 3-month functional outcome and without a quantitative assessment of inter-reader agreement, which may introduce a degree of measurement subjectivity. Fourth, several clinically relevant variables that may influence functional recovery after AIS—including precise stroke subtype/etiology, infarct location and laterality, receipt of intravenous thrombolysis or mechanical thrombectomy, and pre-stroke functional disability—were not available in this retrospective dataset and could not be incorporated into the analysis; time from symptom onset to treatment was recorded in the source medical records but was not extracted for the present analysis. Their absence may have introduced residual confounding and should be addressed in future prospective studies with more comprehensive data capture. Fifth, the same cross-validated predicted probabilities were used both to select the best-performing model and to derive the optimal classification threshold and its associated diagnostic performance indices; this sequential use of the same cross-validation output for model selection and threshold optimization may have introduced a degree of optimism into the reported sensitivity, specificity, and predictive values. Nested cross-validation or bootstrap-based optimism correction would provide a more conservative estimate and is recommended for future work. Sixth, because the study population was restricted to patients who survived to attend the 3-month follow-up visit, patients who died during follow-up were not represented in the analytical sample (no patient had a recorded mRS of 6); this attrition-related selection may have led to an underrepresentation of the most severe cases and could bias the observed associations, and should be considered when interpreting the generalizability of the findings. Finally, as in most observational prognostic analyses, residual confounding factors, such as treatment-related factors, comorbid conditions, and unmeasured clinical variables, were not fully excluded.

## 5. Conclusions

This study demonstrated that the NIHSS score at admission was the strongest independent predictor of poor 3-month functional outcomes after AIS. Lower lesion mean ADC values were associated with poorer outcomes, supporting its value as an imaging marker for more severe acute ischemic injuries, although its adjusted effect remained borderline. Among the blood biomarkers, neutrophil percentage provided the clearest and most consistent prognostic contribution, whereas RDW showed only a borderline signal, and hemoglobin did not contribute an independent predictive value in this cohort. The best-performing model was the base multimarker model combining NIHSS, lesion ADC, neutrophil percentage, and RDW, which achieved the highest cross-validated discrimination among the evaluated models. This suggests that a parsimonious integrated framework may be more useful than either a single-predictor approach or unnecessary expansion of the model with weak variables, such as hemoglobin. Overall, the results of this study support the use of combined clinical, imaging, and inflammatory assessments for early prognostic stratification of AIS. However, larger studies with external validation and, ideally, serial biomarker assessment are needed before such a model can be recommended for routine clinical applications.

## Figures and Tables

**Figure 1:**
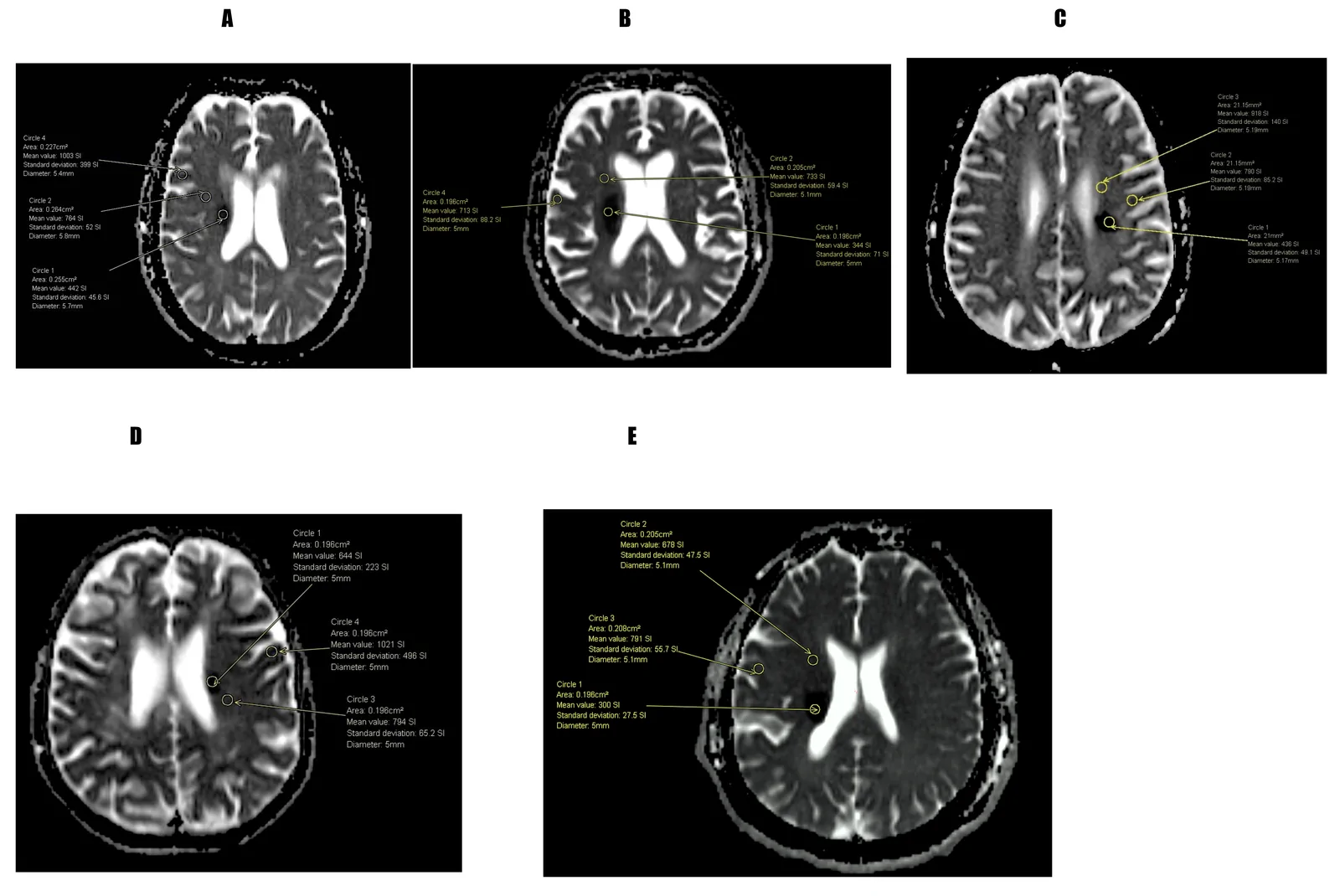
Representative apparent diffusion coefficient (ADC) map images demonstrating region-of-interest (ROI) placement for quantitative ADC measurement in five patients with acute ischemic stroke. Panels (**A**–**E**) show circular ROIs positioned within ischemic lesions to obtain quantitative measurements, including mean ADC, standard deviation, and ROI area. Uniform image dimensions were maintained across panels to facilitate visual comparison.

**Figure 2:**
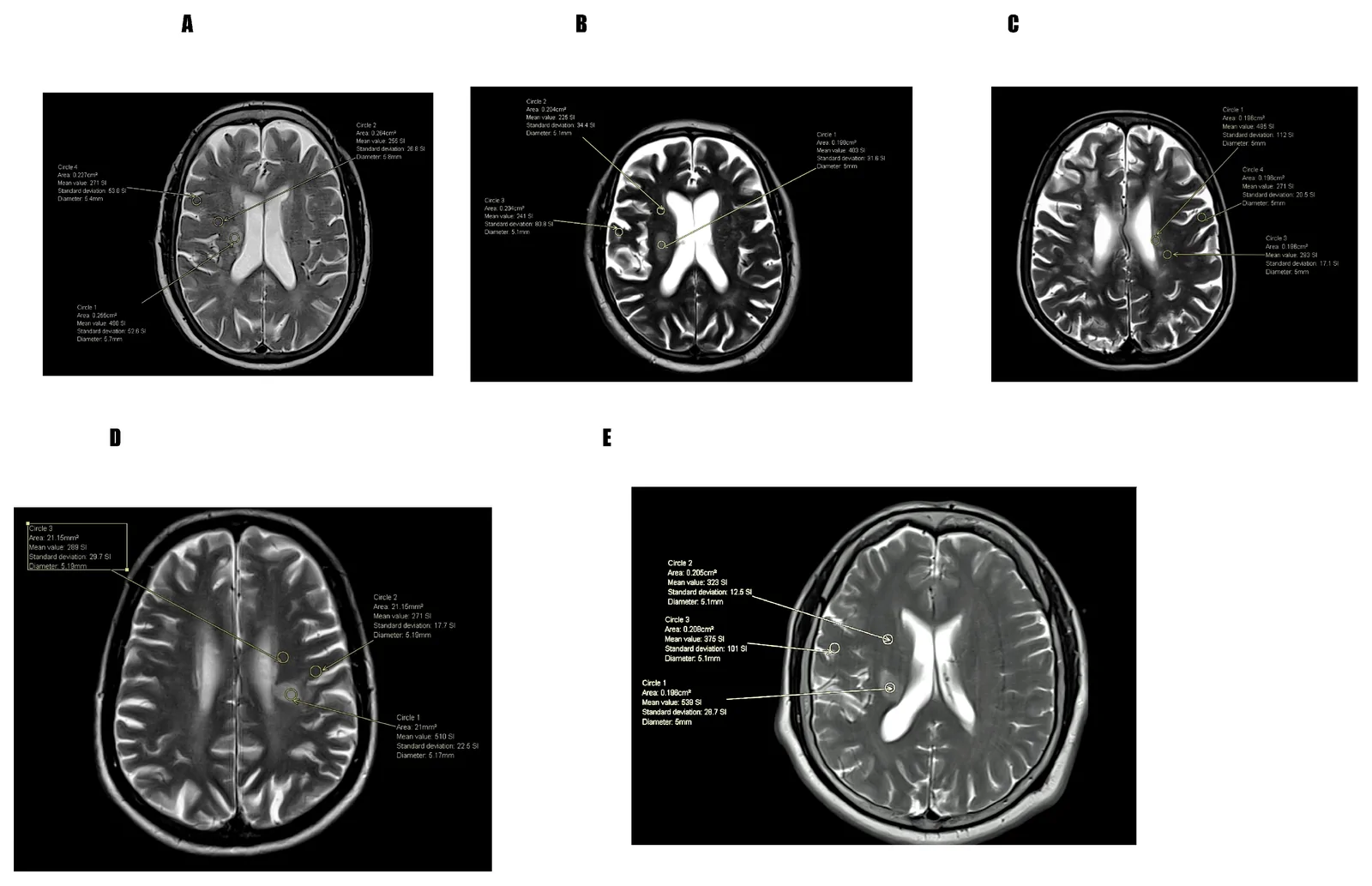
Representative T2-weighted axial magnetic resonance imaging (MRI) images showing region-of-interest (ROI) placement for quantitative T2 signal-intensity analysis in five patients with acute ischemic stroke. Panels (**A**–**E**) show ROIs positioned within ischemic lesions and reference regions in normal-appearing white matter and gray matter. Quantitative measurements included lesion T2 signal intensity and the corresponding reference-tissue values.

**Figure 3:**
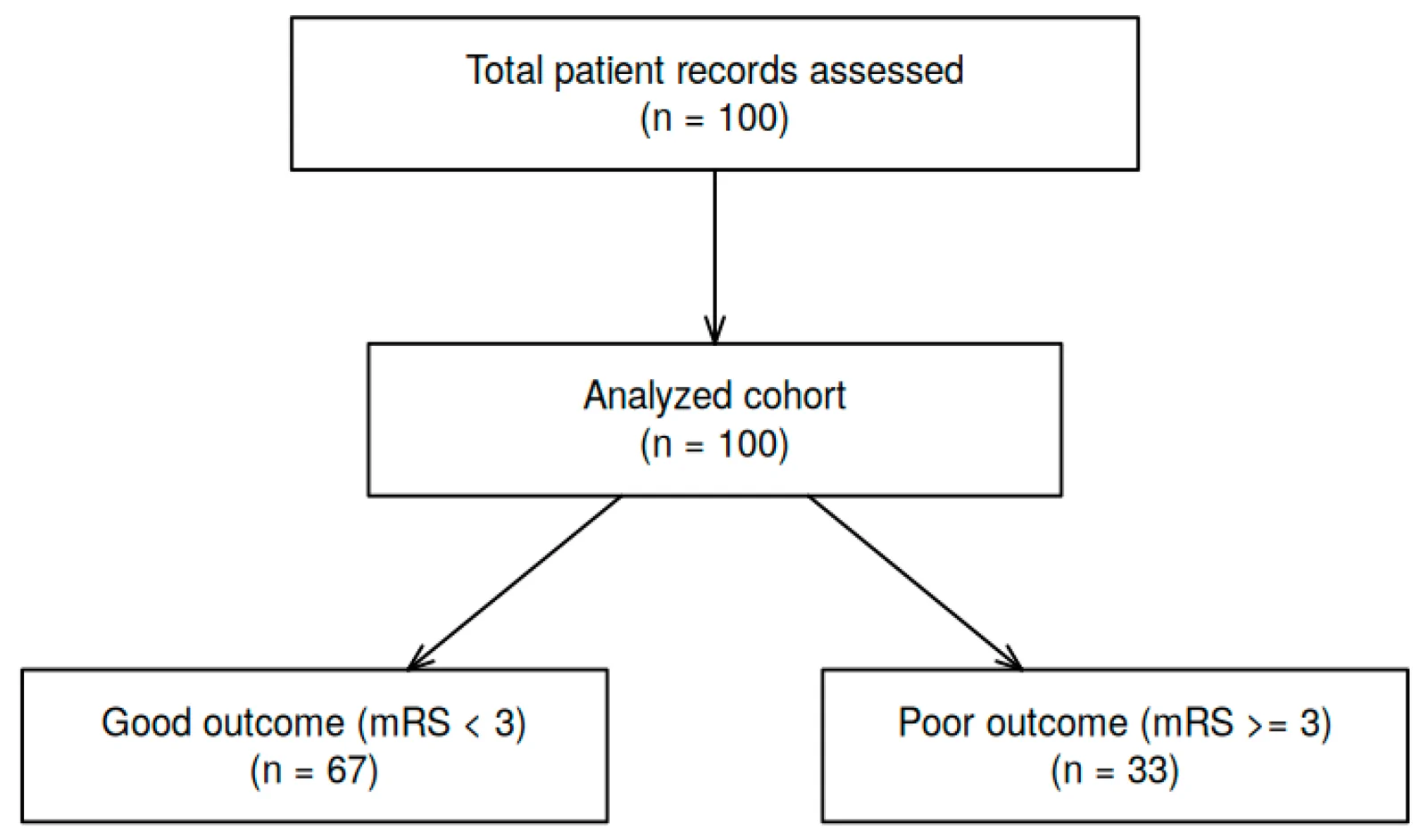
STROBE-style flow diagram of patient selection. Of 100 total patient records assessed, all had valid 3-month modified Rankin Scale (mRS) scores after correction of a single data-entry error, yielding an analyzed cohort of 100 patients (67 with good outcome [mRS < 3] and 33 with poor outcome [mRS ≥ 3]).

**Figure 4:**
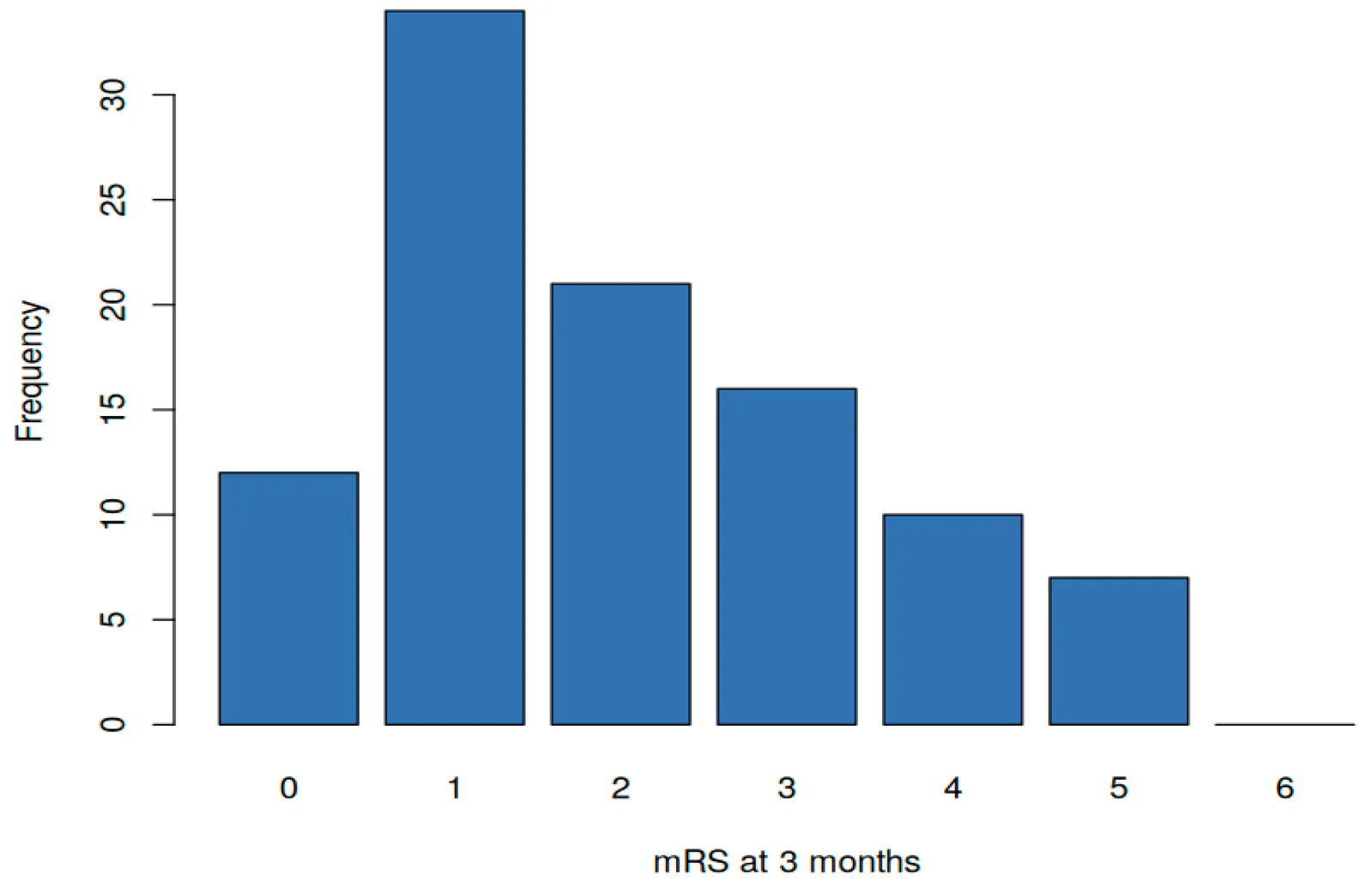
Histogram showing the distribution of 3-month modified Rankin Scale (mRS) scores in the analyzed cohort. The highest frequency was observed at mRS 1, followed by mRS 2 and mRS 0, indicating that most patients achieved favorable functional outcomes at 3 months. The frequency progressively declined across higher mRS categories, whereas mRS 6 was absent.

**Figure 5:**
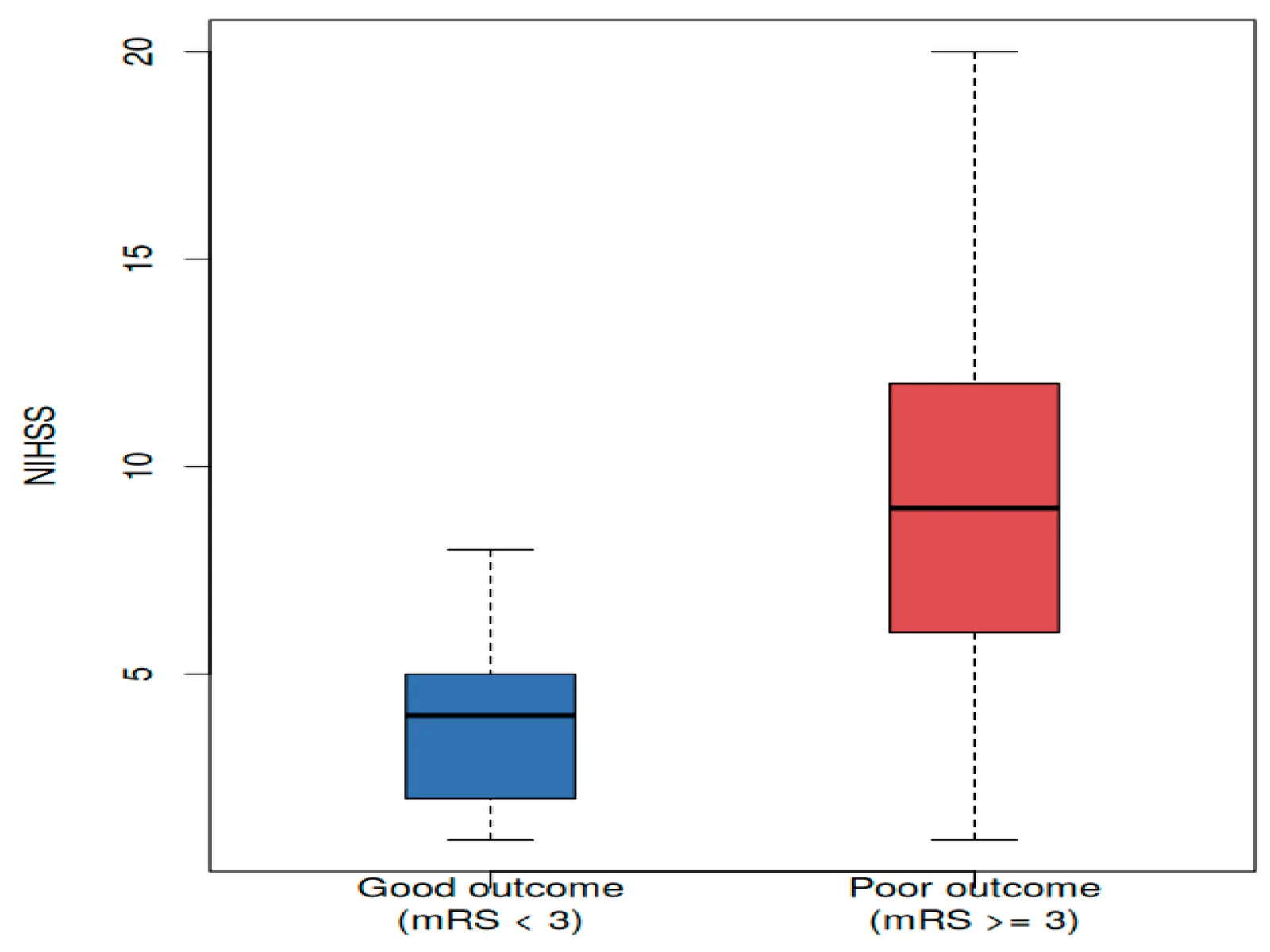
Admission National Institutes of Health Stroke Scale (NIHSS) score by 3-month outcome group. The boxplot shows the distribution of admission NIHSS scores according to 3-month functional outcome. Patients with poor outcomes (mRS ≥ 3) had higher NIHSS values and a wider score distribution than those with good outcomes (mRS < 3).

**Figure 6:**
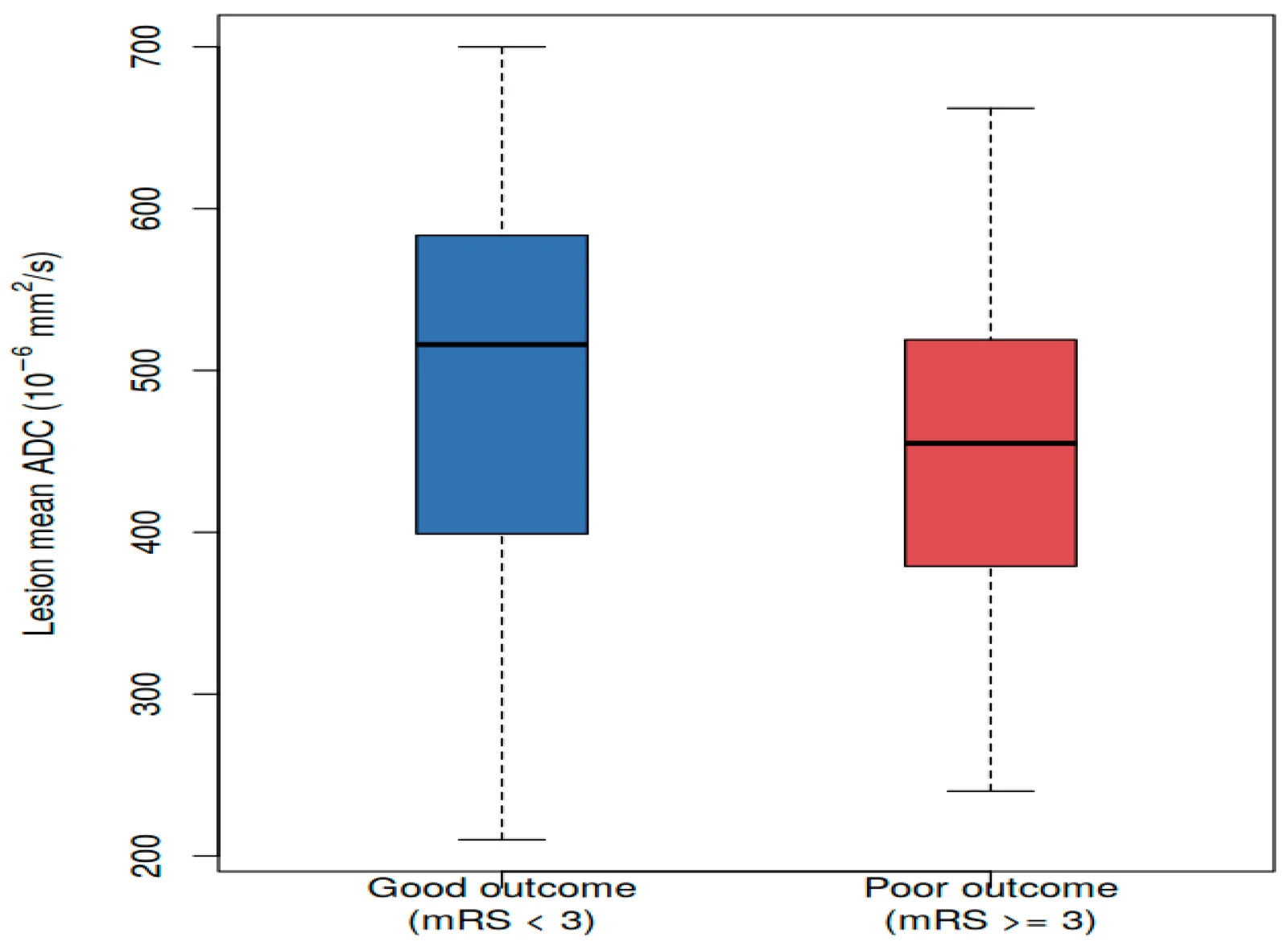
Boxplot showing lesion mean apparent diffusion coefficient (ADC) values by 3-month outcome group (ADC in ×10−6 mm^2^/s). Lower lesion mean ADC values were observed in the poor-outcome group, consistent with stronger diffusion restriction and more severe acute ischemic injury. ADC values were interpreted with reference to the tissue ranges listed in Table 1.

**Figure 7:**
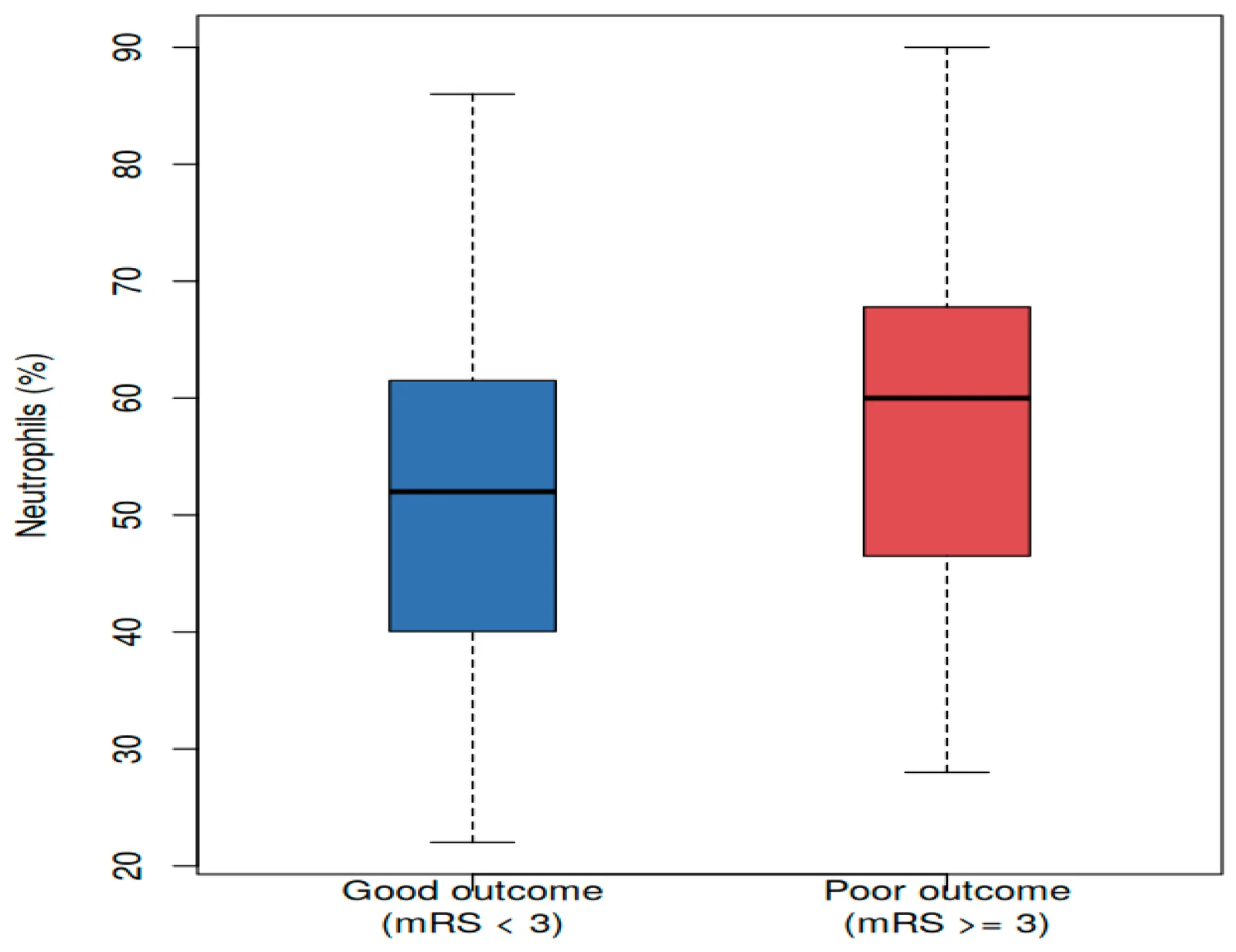
Neutrophil percentage by 3-month outcome group. The boxplot compares neutrophil percentage distributions between outcome groups and visually supports the borderline univariate and significant multivariable inflammatory signal (Table 3, Table 4, Table 6 and Table 7).

**Figure 8:**
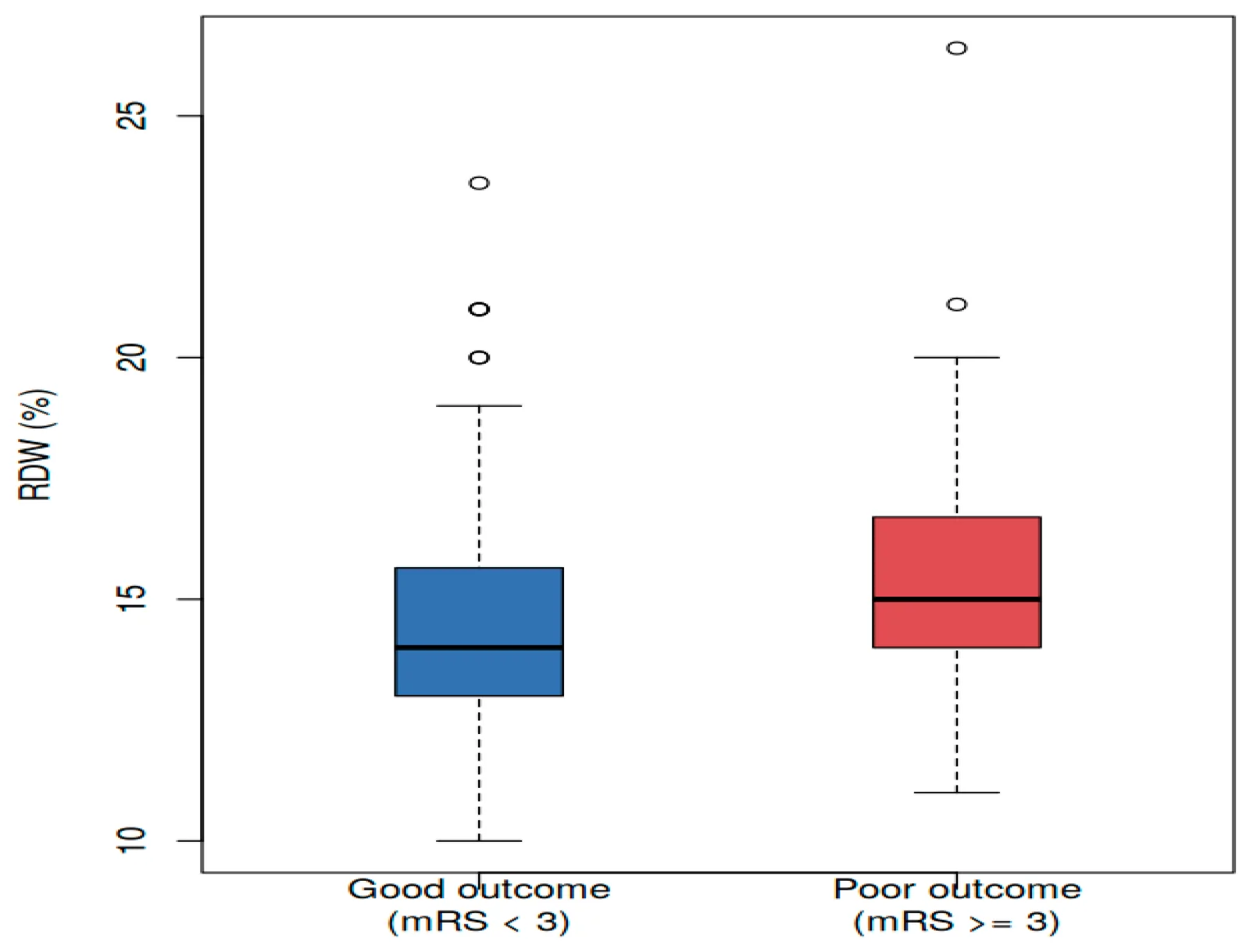
Red cell distribution width (RDW, %) by 3-month outcome group. The boxplot shows partial overlap in RDW distributions between outcome groups, consistent with the borderline univariate difference (*p* = 0.081) and the lack of independent significance after multivariable adjustment (Table 6 and Table 7).

**Figure 9:**
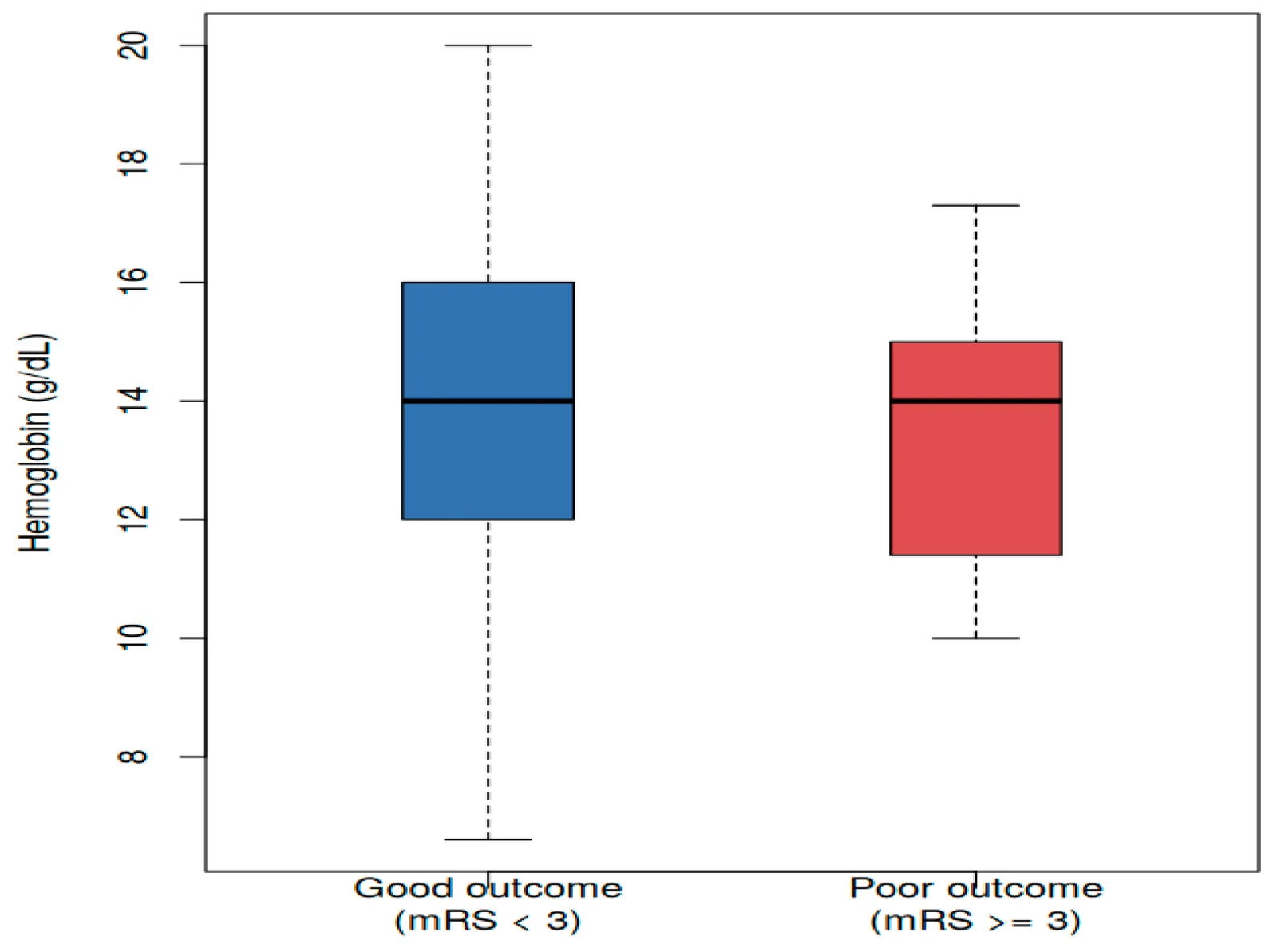
Hemoglobin concentration (g/dL) by 3-month outcome group. The boxplot shows substantial overlap in hemoglobin values between outcome groups, consistent with the non-significant univariate comparison and the absence of an independent effect in the multivariable models.

**Figure 10:**
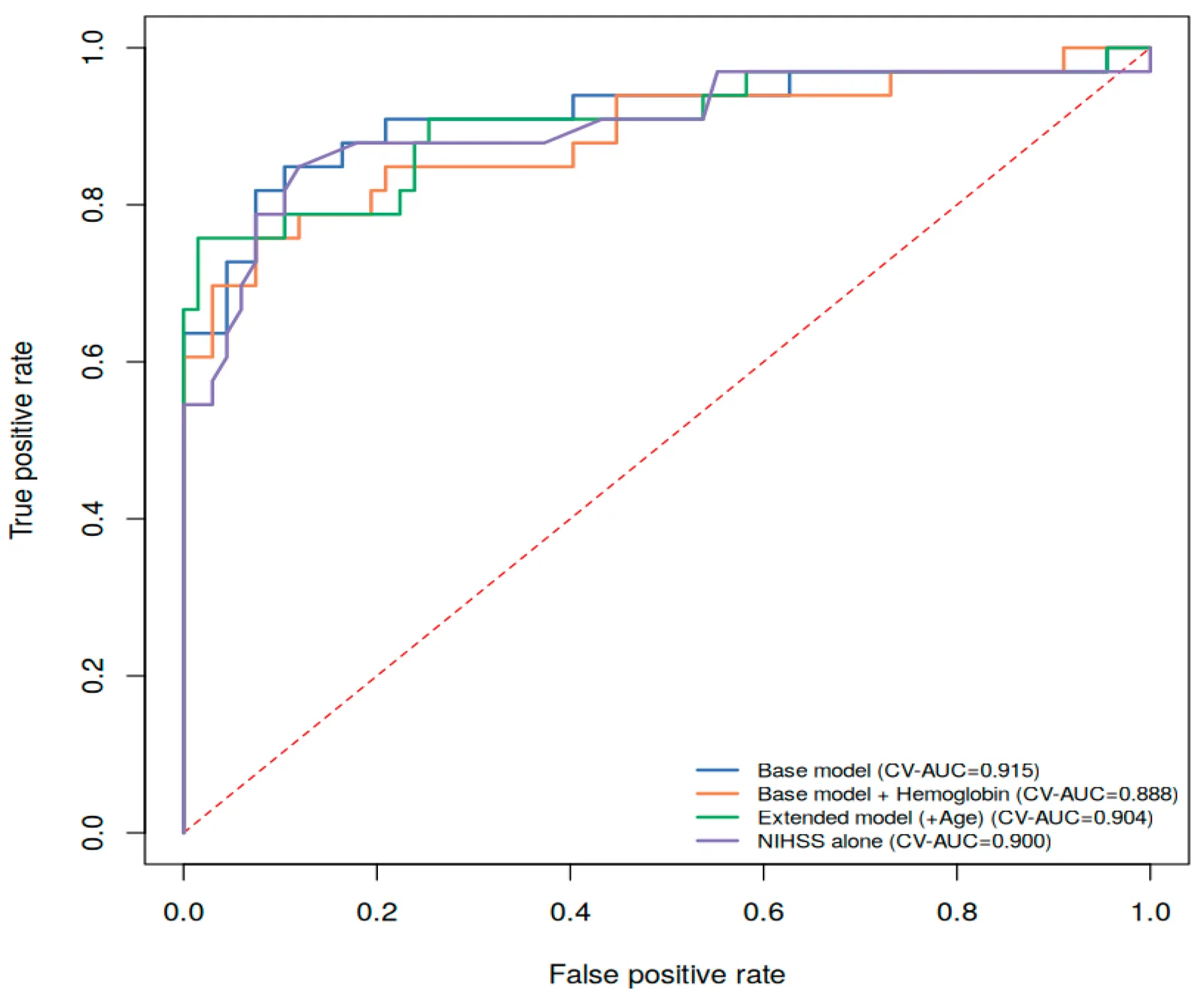
Receiver operating characteristic (ROC) curves for candidate multivariable logistic regression models based on 5-fold stratified cross-validated predicted probabilities. The base model demonstrated the highest cross-validated area under the curve (CV-AUC = 0.906), followed by the hemoglobin-augmented model (CV-AUC = 0.896) and the extended model including age (CV-AUC = 0.892); the National Institutes of Health Stroke Scale (NIHSS)-alone comparator achieved a similar CV-AUC of 0.898. The dashed diagonal line represents chance discrimination (AUC = 0.50).

**Figure 11:**
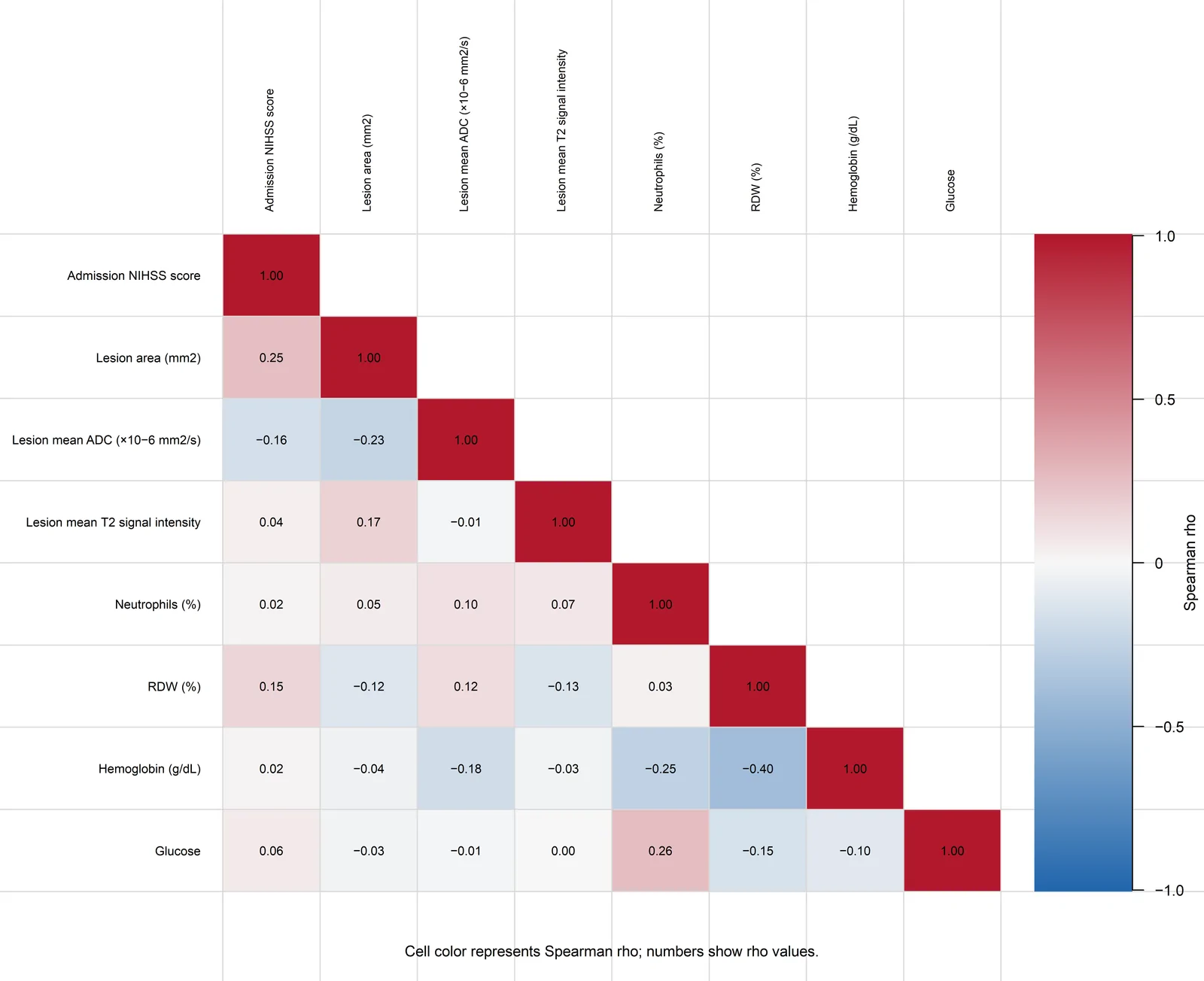
Lower-triangle Spearman correlation heatmap showing pairwise correlation coefficients (rho) among selected clinical, magnetic resonance imaging (MRI), and blood biomarker variables. Darker red shading indicates a larger absolute correlation magnitude, whereas lighter shading indicates weaker correlations; the sign and exact rho value are shown numerically in each cell. The matrix indicates predominantly weak-to-moderate associations and no strong intercorrelations among candidate predictors.

**Figure 12:**
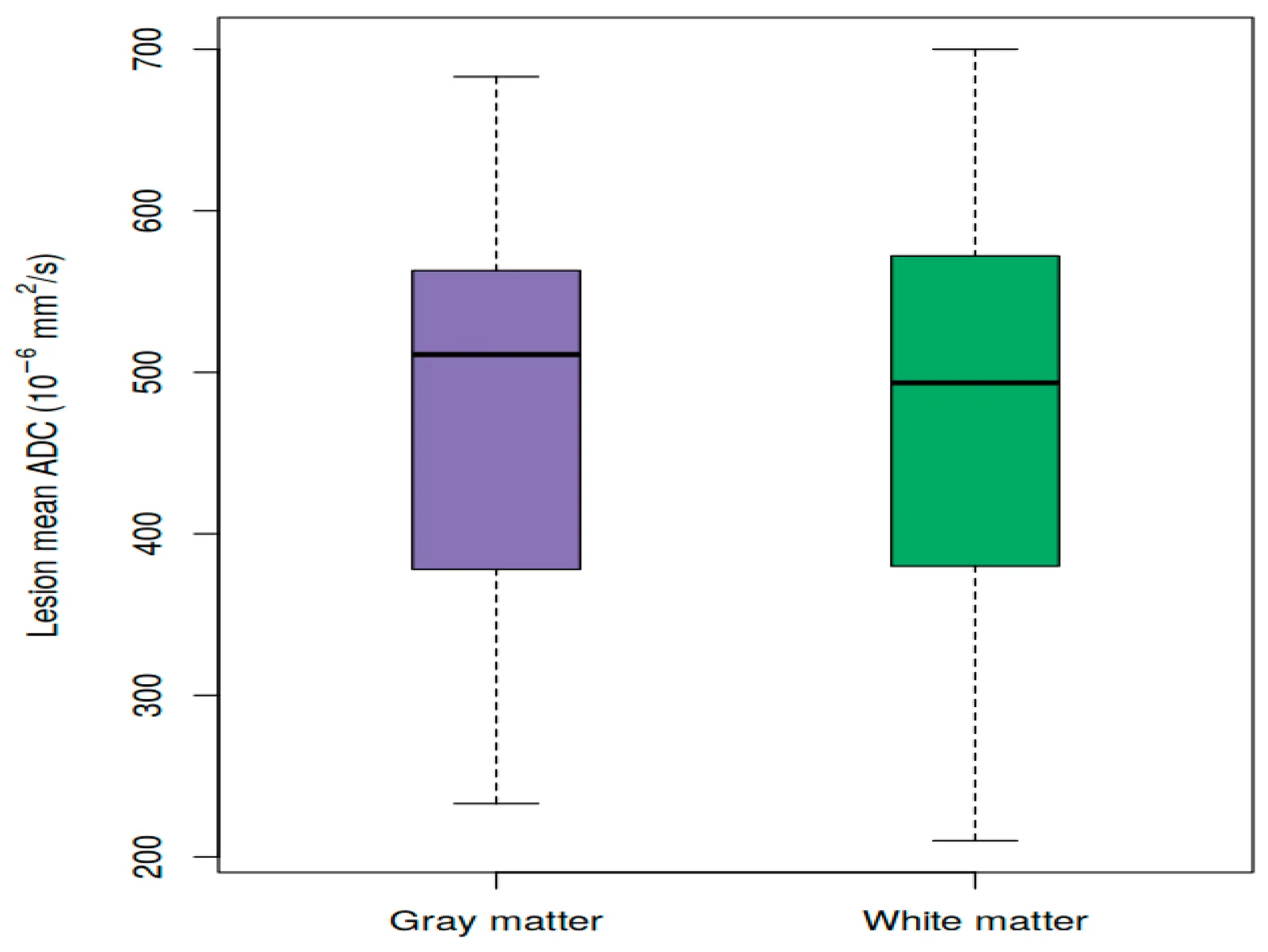
Lesion mean apparent diffusion coefficient (ADC) by lesion location category (gray matter vs. white matter), showing the distribution of lesion mean ADC values according to lesion location and providing anatomical context for lesion-level ADC variability prior to multivariable modeling.

**Figure 13:**
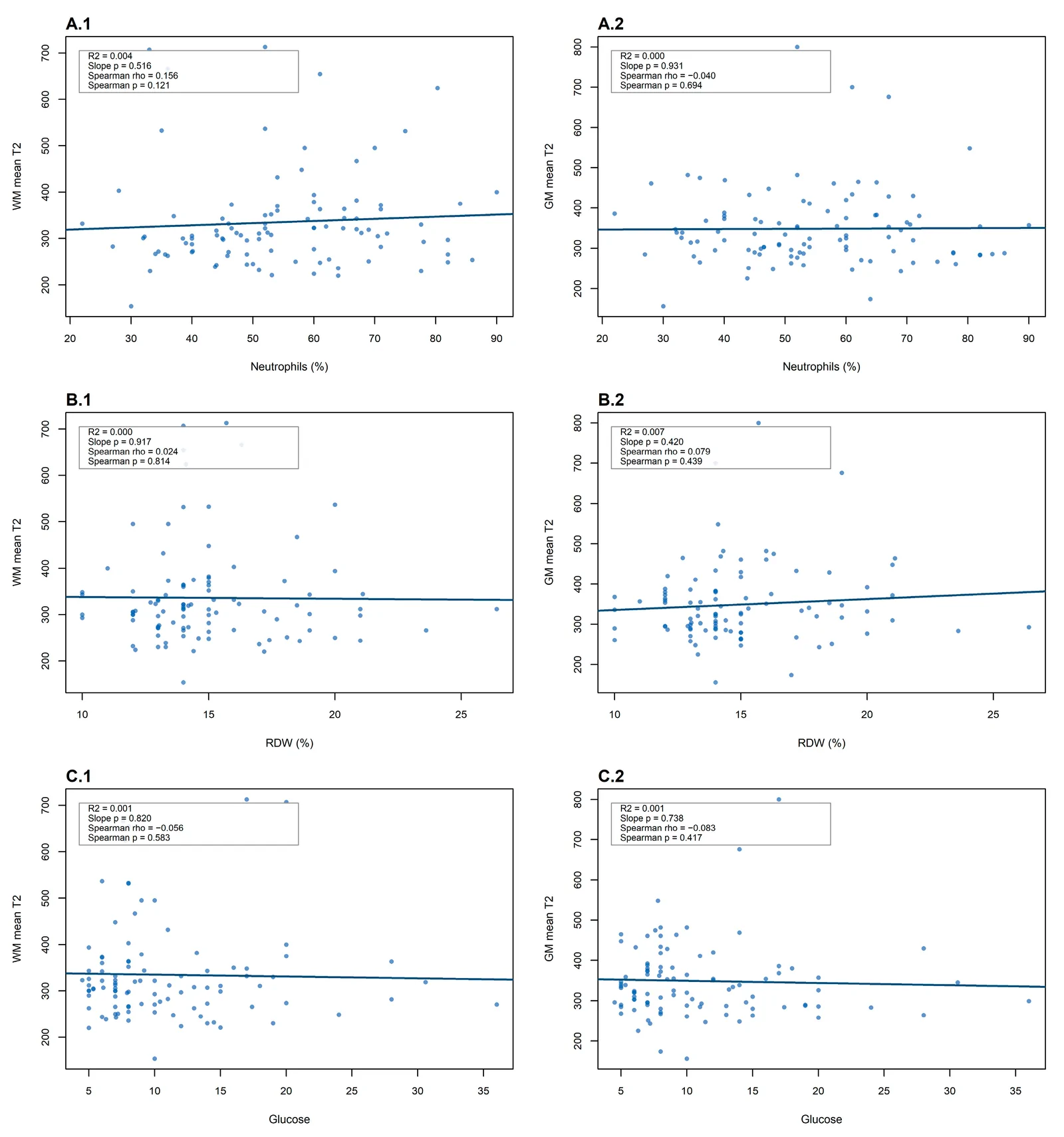
Panel plots of white- and gray-matter T2 signal intensity versus selected blood biomarkers. (**A.1**) white-matter mean T2 versus neutrophil percentage; (**A.2**) gray-matter mean T2 versus neutrophil percentage; (**B.1**) white-matter mean T2 versus red cell distribution width (RDW); (**B.2**) gray-matter mean T2 versus RDW; (**C.1**) white-matter mean T2 versus glucose; (**C.2**) gray-matter mean T2 versus glucose. Each panel includes a fitted linear trendline and in-panel statistics (R^2^, slope *p*-value, Spearman rho, and Spearman *p*-value).

**Figure 14:**
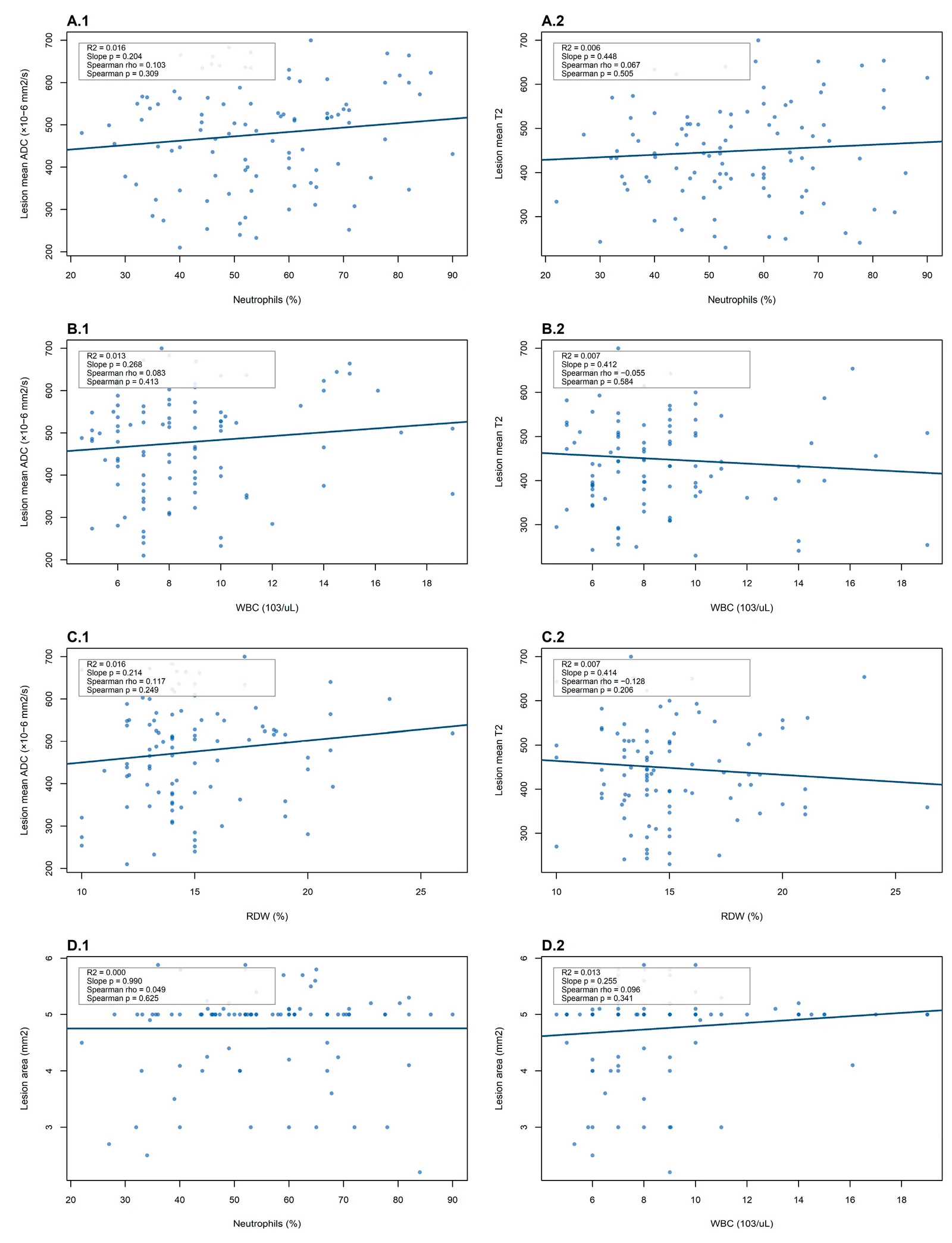
Panel plots of magnetic resonance imaging (MRI) lesion metrics versus inflammatory blood biomarkers. (**A.1**) lesion mean apparent diffusion coefficient (ADC) versus neutrophil percentage; (**A.2**) lesion mean T2 signal intensity versus neutrophil percentage; (**B.1**) lesion mean ADC versus white blood cell count (WBC); (**B.2**) lesion mean T2 signal intensity versus WBC; (**C.1**) lesion mean ADC versus red cell distribution width (RDW); (**C.2**) lesion mean T2 signal intensity versus RDW; (**D.1**) lesion area versus neutrophil percentage; (**D.2**) lesion area versus WBC. Each panel includes a fitted linear trendline and in-panel statistics.

**Figure 15:**
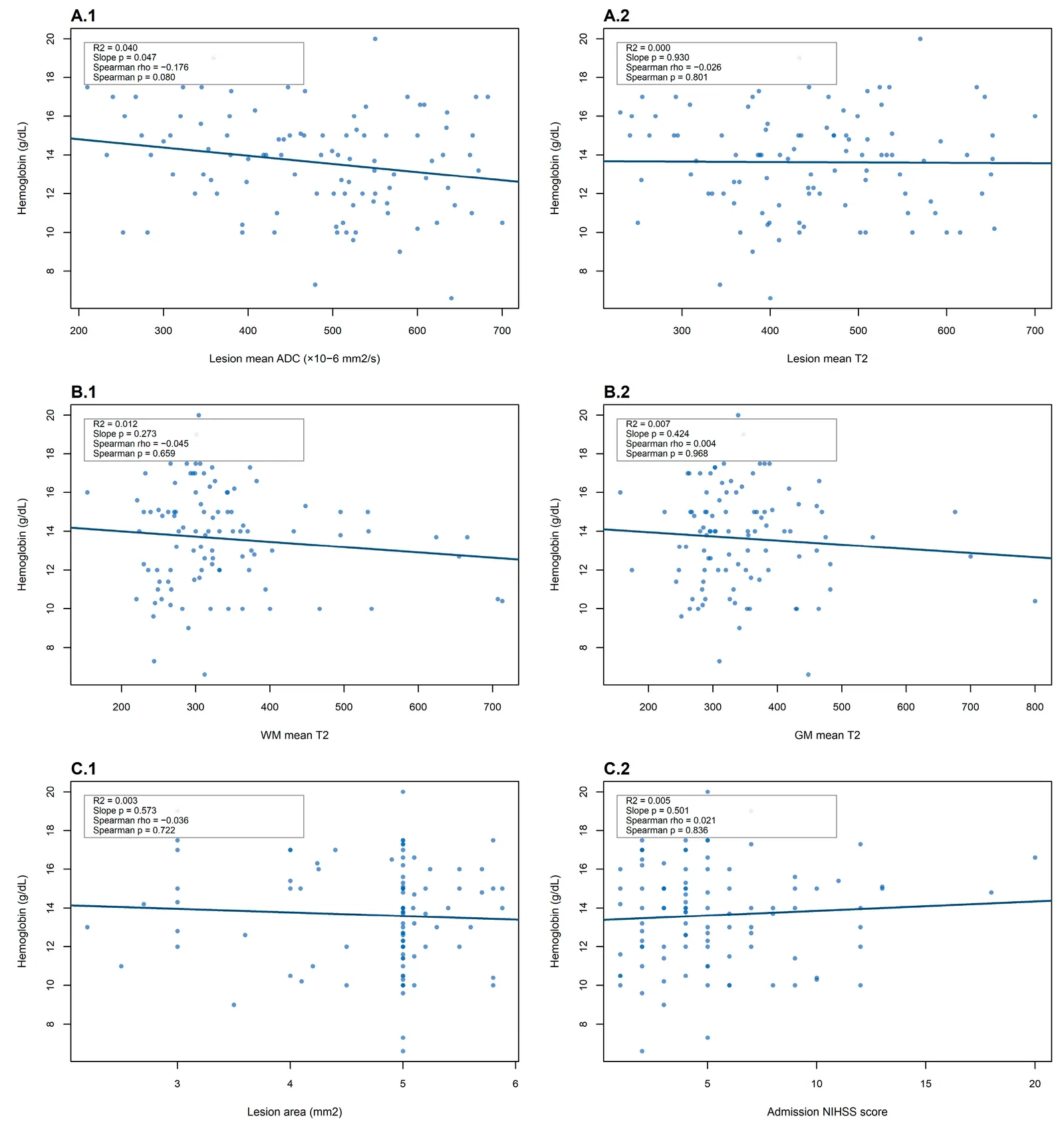
Panel plots of hemoglobin versus magnetic resonance imaging (MRI) lesion metrics and admission National Institutes of Health Stroke Scale (NIHSS) score. (**A.1**) hemoglobin versus lesion mean apparent diffusion coefficient (ADC); (**A.2**) hemoglobin versus lesion mean T2 signal intensity; (**B.1**) hemoglobin versus white-matter mean T2 signal intensity; (**B.2**) hemoglobin versus gray-matter mean T2 signal intensity; (**C.1**) hemoglobin versus lesion area; (**C.2**) hemoglobin versus admission NIHSS score. Each panel includes a fitted linear trendline and in-panel statistics.

**Table 1:** Reference ADC Ranges for MRI Interpretation (×10^−6^ mm^2^/s).

Brain Region	Reference ADC Range (×10^−6^ mm^2^/s)
White matter	670–800
Deep gray matter	700–850
Cortical gray matter	800–1000

ADC, apparent diffusion coefficient; MRI, magnetic resonance imaging. Reference ranges adapted from Helenius et al. (2002) [20].

**Table 2:** Data Quality Summary After Row-Matching Correction.

Item	Value
Total records	100
Records with valid 3-month mRS	100
Good outcome (mRS < 3)	67
Poor outcome (mRS ≥ 3)	33
Invalid/missing mRS excluded	0
Clinical vs. blood age mismatches	None
Clinical vs. blood sex mismatches	0

**Table 3:** Demographic Characteristics of the Analyzed Cohort by 3-Month Functional Outcome.

Variable	Overall (*n* = 100)	Good Outcome (mRS < 3) (*n* = 67)	Poor Outcome (mRS ≥ 3) (*n* = 33)	*p*-Value
Age, years	61.0 (50.8–74.0)	59.0 (50.0–70.5)	65.0 (51.0–80.0)	0.165
Male sex, *n* (%)	75 (75.0)	51 (76.1)	24 (72.7)	0.807
Female sex, *n* (%)	25 (25.0)	16 (23.9)	9 (27.3)	0.807

**Table 4:** Baseline Clinical, MRI, and Blood Biomarker Variables in Good- and Poor-Outcome Groups.

Variable	Good Outcome (mRS < 3)	Poor Outcome (mRS ≥ 3)	*p*-Value
Age (years)	59.0 (50.0–70.5)	65.0 (51.0–80.0)	0.165
Admission NIHSS score	4.0 (2.0–5.0)	9.0 (6.0–12.0)	<0.001
Lesion area (mm^2^)	5.0 (4.2–5.0)	5.0 (5.0–5.1)	0.078
Lesion mean ADC (×10^−6^ mm^2^/s)	516.0 (399.0–583.5)	455.0 (379.0–519.0)	0.072
Lesion ADC SD (×10^−6^ mm^2^/s)	71.3 (44.3–100.5)	50.8 (38.5–77.3)	0.112
Lesion mean T2 signal intensity	433.0 (370.5–525.0)	485.0 (397.0–526.0)	0.337
Neutrophils (%)	52.0 (40.0–61.5)	60.0 (46.5–67.8)	0.074
Lymphocytes (%)	36.0 (26.1–46.5)	31.0 (23.0–43.0)	0.242
RDW (%)	14.0 (13.0–15.7)	15.0 (14.0–16.4)	0.081
Hemoglobin (g/dL)	14.0 (12.0–16.0)	14.0 (11.4–15.0)	0.386
Glucose (mmol/L)	8.0 (7.0–13.5)	9.1 (7.0–13.9)	0.253
INR	1.0 (0.9–1.1)	1.0 (0.9–1.1)	0.625
APTT (seconds)	30.0 (27.0–33.0)	29.0 (28.0–32.0)	0.950

MRI—magnetic—resonance imaging. The variables were compared at 3 months. Values are presented as median (IQR). Continuous variables were compared using the Mann–Whitney–U test. Good outcome = mRS < 3; poor outcome = mRS ≥ 3. Statistical significance was defined as *p* < 0.05 (two-sided). ADC values are reported in units of ×10^−6^ mm^2^/s.

**Table 5:** Ranked Univariate Comparison of Clinical, MRI, and Blood Biomarker Variables.

Rank	Variable	Good Outcome (mRS < 3)	Poor Outcome (mRS ≥ 3)	*p*-Value
1	Admission NIHSS score	4.0 (2.0–5.0)	9.0 (6.0–12.0)	<0.001
2	Lesion location (white matter = 1)	1.0 (0.0–1.0)	1.0 (1.0–1.0)	0.060
3	Lesion mean ADC (×10^−6^ mm^2^/s)	516.0 (399.0–583.5)	455.0 (379.0–519.0)	0.072
4	Neutrophils (%)	52.0 (40.0–61.5)	60.0 (46.5–67.8)	0.074
5	Lesion area (mm^2^)	5.0 (4.2–5.0)	5.0 (5.0–5.1)	0.078
6	RDW (%)	14.0 (13.0–15.7)	15.0 (14.0–16.4)	0.081
7	Lesion ADC SD (×10^−6^ mm^2^/s)	71.3 (44.3–100.5)	50.8 (38.5–77.3)	0.112
8	Age (years)	59.0 (50.0–70.5)	65.0 (51.0–80.0)	0.165
9	White matter mean T2 signal intensity	306.0 (266.5–345.5)	322.0 (282.0–372.0)	0.203
10	Lymphocytes (%)	36.0 (26.1–46.5)	31.0 (23.0–43.0)	0.242
11	White matter T2 signal intensity SD	23.1 (16.9–33.2)	25.9 (20.1–35.4)	0.267
12	RBC count (×10^6^/µL)	5.5 (5.0–6.0)	6.0 (5.0–6.0)	0.349
13	Hemoglobin (g/dL)	14.0 (12.0–16.0)	14.0 (11.4–15.0)	0.386
14	Gray matter mean T2 signal intensity	325.0 (286.5–376.5)	334.0 (299.0–392.0)	0.393

MRI—magnetic—resonance imaging. The variables were compared between good- and poor-outcome groups at 3 months. Variables are ranked according to ascending univariate *p*-value. Data are presented as median (interquartile range, IQR). Continuous variables were compared using the Mann–Whitney–U test. Functional outcome was classified according to the 3-month modified Rankin Scale (mRS) as good outcome (mRS < 3) and poor outcome (mRS ≥ 3). A two-sided *p*-value < 0.05 was considered statistically significant. ADC values are reported in units of ×10^−6^ mm^2^/s.

**Table 6:** Predictive Performance of Multivariable Logistic Regression Models (Apparent AUC and CV-AUC).

Model	Predictor Set	Apparent AUC	CV-AUC
Base model	Admission NIHSS score; Lesion mean ADC (×10^−6^ mm^2^/s); Neutrophils (%); RDW (%)	0.934	0.906
Base model + hemoglobin	Admission NIHSS score; Lesion mean ADC (×10^−6^ mm^2^/s); Neutrophils (%); RDW (%); Hemoglobin (g/dL)	0.932	0.896
Extended model	Age (years); Admission NIHSS score; Lesion mean ADC (×10^−6^ mm^2^/s); Neutrophils (%); RDW (%); Hemoglobin (g/dL)	0.937	0.892
NIHSS alone (comparator)	Admission NIHSS score	0.906	0.898

AUC—area under the curve—; CV-AUC—cross-validated AUC—. Apparent AUC = area under the receiver operating characteristic (ROC) curve calculated on the fitted dataset. CV-AUC = mean AUC based on 5-fold stratified cross-validation. Higher CV-AUC indicates better out-of-sample discrimination.

**Table 7:** Multivariable Logistic Regression Estimates for the Base Model.

Predictor	OR	95% CI	*p*-Value
Admission NIHSS score	2.57	1.70–3.87	<0.001
Lesion mean ADC (×10^−6^ mm^2^/s)	0.99	0.99–1.00	0.069
Neutrophils (%)	1.07	1.02–1.12	0.010
RDW (%)	1.12	0.91–1.38	0.278

OR = odds ratio; CI = confidence interval. Odds ratios were estimated from a multivariable logistic regression model for poor 3-month functional outcome (mRS ≥ 3). *p*-values are two-sided.

**Table 8:** Multivariable Logistic Regression Estimates for the Base Model Plus Hemoglobin.

Predictor	OR	95% CI	*p*-Value
Admission NIHSS score	2.55	1.69–3.85	<0.001
Lesion mean ADC (×10^−6^ mm^2^/s)	0.99	0.99–1.00	0.066
Neutrophils (%)	1.06	1.01–1.12	0.022
RDW (%)	1.10	0.88–1.38	0.382
Hemoglobin (g/dL)	0.94	0.68–1.30	0.719

OR = odds ratio; CI = confidence interval. Odds ratios were estimated from a multivariable logistic regression model for poor 3-month functional outcome (mRS ≥ 3) after adding hemoglobin to the base model. *p*-values are two-sided.

**Table 9:** ROC-Derived Optimal Probability Cutoff Metrics for the Best-Performing Cross-Validated Model.

Metric	Value
Best-performing model (by CV-AUC)	Base model (NIHSS + ADC + Neutrophils + RDW)
CV-AUC	0.906
Optimal probability cutoff (Youden index)	0.382
Sensitivity	81.8%
Specificity	89.6%
Positive predictive value (PPV)	79.4%
Negative predictive value (NPV)	90.9%
Accuracy	87.0%

ROC—receiver operating characteristic. The optimal probability cutoff was selected using the Youden index from the ROC curve of the best-performing model based on 5-fold stratified cross-validated predicted probabilities. PPV = positive predictive value; NPV = negative predictive value.

**Table 10:** Top MRI-Blood Spearman Correlations Ranked by *p*-Value, With Strength-Category Star Notation.

Rank	MRI/Clinical Variable	Blood Biomarker	N	Spearman Rho	Strength Stars	*p*-Value	R^2^ (Linear Trend)	FDR q-Value
1	Lesion ADC SD (×10^−6^ mm^2^/s)	INR	100	0.267	* (weak)	0.007	0.013	0.453
2	Lesion mean ADC (×10^−6^ mm^2^/s)	INR	100	0.216	* (weak)	0.031	0.021	0.502
3	Lesion ADC SD (×10^−6^ mm^2^/s)	Hemoglobin (g/dL)	100	−0.207	* (weak)	0.039	0.000	0.502
4	Lesion mean T2 signal intensity	INR	100	−0.200	* (weak)	0.046	0.006	0.502
5	Lesion ADC SD (×10^−6^ mm^2^/s)	RDW (%)	99	0.201	* (weak)	0.046	0.011	0.502
6	Gray matter mean T2 signal intensity	APTT (seconds)	100	−0.188	ns (very weak/negligible)	0.062	0.027	0.502
7	Lesion ADC SD (×10^−6^ mm^2^/s)	Lymphocytes (absolute count)	100	−0.186	ns (very weak/negligible)	0.064	0.030	0.502
8	Lesion ADC SD (×10^−6^ mm^2^/s)	Neutrophils (absolute count)	100	0.186	ns (very weak/negligible)	0.064	0.032	0.502
9	Admission NIHSS score	WBC (10^3^/µL)	100	0.179	ns (very weak/negligible)	0.074	0.008	0.502
10	Lesion mean ADC (×10^−6^ mm^2^/s)	Hemoglobin (g/dL)	100	−0.176	ns (very weak/negligible)	0.080	0.040	0.502
11	Lesion mean T2 signal intensity	Platelets (10^3^/µL)	100	−0.158	ns (very weak/negligible)	0.117	0.011	0.607
12	White matter mean T2 signal intensity	Neutrophils (absolute count)	100	0.156	ns (very weak/negligible)	0.121	0.004	0.607
13	Gray matter mean T2 signal intensity	Lymphocytes (absolute count)	100	0.154	ns (very weak/negligible)	0.125	0.004	0.607
14	Admission NIHSS score	RDW (%)	99	0.151	ns (very weak/negligible)	0.136	0.010	0.610
15	White matter mean T2 signal intensity	WBC (10^3^/µL)	100	0.146	ns (very weak/negligible)	0.147	0.034	0.616
16	White matter mean T2 signal intensity	INR	100	−0.136	ns (very weak/negligible)	0.177	0.001	0.697
17	Lesion mean T2 signal intensity	RDW (%)	99	−0.128	ns (very weak/negligible)	0.206	0.007	0.713
18	Lesion area (mm^2^)	APTT (seconds)	100	−0.126	ns (very weak/negligible)	0.211	0.036	0.713
19	Lesion ADC SD (×10^−6^ mm^2^/s)	WBC (10^3^/µL)	100	0.124	ns (very weak/negligible)	0.219	0.038	0.713
20	Lesion area (mm^2^)	RDW (%)	99	−0.120	ns (very weak/negligible)	0.236	0.006	0.713

* = weak (0.20 ≤ |rho| < 0.40); ns = very weak/negligible (|rho| < 0.20). R^2^ values represent fitted linear trendlines in exploratory scatterplots and are provided as descriptive visual aids only; inferential interpretation is based on Spearman rho and the corresponding *p*-value. After Benjamini–Hochberg correction across the full family of 63 pairwise MRI/clinical–blood biomarker comparisons actually tested (including the hemoglobin-specific comparisons reported separately in Table 11), none of the associations remained statistically significant (all FDR q-values > 0.05); nominal *p*-values should therefore be interpreted as hypothesis-generating rather than confirmatory.

**Table 11:** Hemoglobin-Specific Correlations With MRI and Clinical Severity Variables, With Strength-Category Star Notation.

Rank	Comparator Variable	N	Spearman Rho	Strength Stars	*p*-Value	R^2^	FDR q-Value
1	Lesion ADC SD (×10^−6^ mm^2^/s)	100	−0.207	* (weak)	0.039	0.000	0.502
2	Lesion mean ADC (×10^−6^ mm^2^/s)	100	−0.176	ns (very weak/negligible)	0.080	0.040	0.502
3	White matter mean T2 signal intensity	100	−0.045	ns (very weak/negligible)	0.659	0.012	0.933
4	Lesion area (mm^2^)	100	−0.036	ns (very weak/negligible)	0.722	0.003	0.958
5	Lesion mean T2 signal intensity	100	−0.026	ns (very weak/negligible)	0.801	0.000	0.964
6	Admission NIHSS score	100	0.021	ns (very weak/negligible)	0.836	0.005	0.964
7	Gray matter mean T2 signal intensity	100	0.004	ns (very weak/negligible)	0.968	0.007	0.988

MRI—magnetic resonance imaging; * = weak (0.20 ≤ |rho| < 0.40); ns = very weak/negligible (|rho| < 0.20). Strength notation describes effect-size magnitude and does not replace formal significance testing. R^2^ values are descriptive and correspond to fitted linear trendlines in the accompanying scatterplots. FDR q-values for these 7 hemoglobin-specific comparisons were computed jointly with the 56 comparisons in Table 10 as a single family of 63 pairwise tests (Benjamini–Hochberg correction); none remained statistically significant (all FDR q-values > 0.05).

## Data Availability

The data that supports the findings of this study are available upon request.

## Abbreviations

AIS	Acute ischemic stroke
PPV	Positive predictive value
NPV	Negative predictive value
ROC	Receiver operating characteristic
AUC	Area under the curve
RBC	Red blood cell
mRS	modified Rankin Scale
ADC	apparent diffusion coefficient
GFAP	Glial fibrillary acidic protein
NfL	Neurofilament light chain
NSE	Neuron-specific enolase
UCH-L1	Ubiquitin Carboxy-terminal Hydrolase L1
MMP-9	Matrix Metalloproteinase-9
RDW	Red cell distribution width
SD	Standard deviation
NIHSS	National Institutes of Health Stroke Scale
FLAIR	Fluid-Attenuated Inversion Recovery
